# Synergistic Se‐Doping and Accompanying S‐Vacancies Drive Lattice Distortion and p‐Band Modulation for Enhanced Sodium Storage in Sulfides

**DOI:** 10.1002/advs.75070

**Published:** 2026-04-02

**Authors:** Chunyang Xu, Jinfeng Liu, Biao Ma, Yawen Liang, Dongdong Wang, Chengcheng Miao, Jianhua Zhu, Liu Wang, Yunfeng Chao, Xinwei Cui

**Affiliations:** ^1^ Henan Institute of Advanced Technology Zhengzhou University Zhengzhou P. R. China; ^2^ Qinghai Huanghe Hydropower Development Co., Ltd. Xining Qinghai P. R. China; ^3^ College of Energy Engineering Huanghuai University Zhumadian Henan P. R. China; ^4^ Henan Great Power Energy Co. Ltd. Zhumadian Henan P. R. China

**Keywords:** p‐band center, Se doping, S vacancies, sodium‐ion batteries, sulfides

## Abstract

Metal sulfides, especially those containing alloying‐capable metal elements, have shown great potential as sodium storage materials. However, the complex reaction process imposes more stringent requirements on the kinetic rate. A simple lattice distortion strategy is first proposed in this work to engineer the p‐band center of S in Cu_3_SbS_3_ for enhanced Na^+^ storage. Though the co‐doping of Se and accompanying S vacancies, the resultant lattice distortion elongated the bonds between metal and nonmetal elements to weaken their orbital hybridization and lift the p‐band center for a high Na^+^ adsorption. The defects also increase the number of charge carriers for an improved conductivity. All those merits combined to contribute a superior electrochemical reaction kinetic in the Se‐Cu_3_SbS_3_@rGO anode. It achieved a high capacity of 569.1 mAh g^−1^ at 0.1 A g^−1^, excellent rate capability of 273.6 mAh g^−1^ at 50 A g^−1^ and superior cycling stability of 387.4 mAh g^−1^ (90%) after 1000 cycles at 5 A g^−1^ as well as 92% after 150 cycles at 0.5 A g^−1^ in the full cells. The insights into the function mechanism of Se‐vacancies doping‐induced lattice distortion offer valuable insights into the design and development of sulfide‐based anode materials and sodium‐ion batteries.

## Graphical abstract

Se‐doping in sulfides facilitates the creation of S vacancies. The collaboration of Se‐doping and S vacancies not only induces lattice distortion to weaken the M─S bonds and lift the p‐band center for improved Na^+^ adsorption, but also increases the number of charge carriers for an improved electronic conductivity. The resultant Se‐Cu_3_SbS_3_@rGO achieved excellent reaction kinetics and performance.

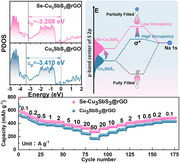

## 1 Introduction

With the rapid development of clean energy technologies, lithium‐ion batteries (LIBs) have achieved a dominant position in numerous applications such as electric vehicles and unmanned aerial systems [1, 2]. However, challenges such as the uneven distribution of lithium resources, significant price volatility, and safety concerns still hinder their widespread deployment. Sodium‐ion batteries (SIBs), owing to their abundant reserves, appropriate redox potential, and similar working mechanisms to LIBs, have been widely regarded as a promising complementary energy storage system [3, 4, 5]. Nevertheless, the larger ionic radius of Na^+^ (1.02 Å) leads to sluggish reaction kinetics, poor reversibility, and pronounced volume expansion, thereby imposing stricter requirements on anode materials [6, 7]. Among various candidates, metal sulfides (MS_x_) have attracted significant attention due to their low cost, facile synthesis, and high theoretical capacity [1, 8]. Specifically, bimetallic sulfides were highlighted for their superior electrochemical kinetics and higher density of active sites [9, 10, 11]. Some p‐block metal‐based sulfides can offer additional advantages by contributing capacity through both conversion and alloying reactions [12]. For example, Cu_3_SbS_3_ can deliver a high theoretical capacity of ∼590 mAh g^−1^ through a combination of conversion and alloying reactions [13]. However, the complex electrochemical process also sets a high requirement on the reaction kinetics to ensure a good reversibility.

Introducing carbon materials and constructing porous architectures were the two common strategies that developed to address those challenges [14, 15, 16]. Benefited by the high electronic conductivity of carbon and the large surface area of porous structures, transport of electrons and ions in the electrodes can be well facilitated. However, the intrinsic issues of metal sulfides had not been fundamentally resolved. In MS_x_ materials, the electronic structure of sulfur atoms plays a decisive role in Na^+^ adsorption and the occurrence of conversion reactions [17]. Recent studies have shown the effectiveness of modulating the p‐band center of non‐metallic elements to enhance their cation adsorption capabilities [18, 19]. Namely, elevating the p‐band center of sulfur can be critical for the Na^+^ adsorption in MS_x_. Several approaches have been reported to regulate the p‐band center of non‐metallic elements, including ion intercalation [20], vacancy creation [21], heteroatom doping [22], and spin‐state engineering [17]. For instance, the introduction of transition metal atoms (Cu, Mg, Co, et al.) into MnO_2_ modifies the Mn─O bond environment and tunes the O p‐band center [22]. The spin polarization and S vacancy can lift the p‐band center of S and weaken the Fe─S bonds in FeS_2_ [21]. Cu‐induced spin‐state regulation of Co weakens the Co─Se bond, which enhances the p‐band center of Se and promotes Na^+^ adsorption [17].

Inspired by the recent advancements in lattice distortion engineering [23, 24, 25], a novel approach was proposed in this work to regulate the sulfur p‐band center in sulfides for enhanced Na^+^ adsorption and diffusion, as well as exploring the potentials of lattice distortion modulation. Recent research has shown that lattice distortion is a powerful route of tuning the d‐band center in transition metals. For example, in P─CoSe_2_/MXene systems, P‐doping induced lattice distortion increases the Co d‐band center, leading to improved adsorption and catalytic conversion of polysulfides [24]. Therefore, regulating lattice distortion to weaken the M─S bonds can be potentially elevate the p‐band center. It was reported that lattice distortion can be induced via elemental substitution or vacancy doping. Notably, doping atoms with larger sizes or low electronegativity can simultaneously introduce accompanying vacancies [26, 27]. From this view, Se can be a suitable doping element for sulfides. Introducing Se into Cu_3_SbS_3_ can simultaneously realize elemental substitution and vacancies creation to achieve lattice distortion, therefore, achieving an increased p‐band center for a rapid reaction kinetic.

In this study, Se‐doped Cu_3_SbS_3_ nanospheres encapsulated in a carbon shell and anchored on a reduced graphene oxide (rGO) substrate were synthesized via a simple hydrothermal method. The rGO and carbon shell ensure excellent electrical conductivity, good electrolyte accessibility, and accommodation of the volume changes. More importantly, the doped Se atoms successfully induced the formation of S vacancies. They combined to cause lattice distortion and elongated bonds between metal and non‐metal elements. The resultant electronic redistribution and weakened orbital hybridization and also elevates the S p‐band center for better Na^+^ adsorption. In addition, the introduction of these defects also provides more charge carriers for a higher electronic conductivity. Eventually, the produced Se‐Cu_3_SbS_3_@rGO obtained superior electrochemical reaction kinetics and sodium storage performance, which not only demonstrates the effectiveness of this Se‐vacancies doping strategy, but also provides new insights and theoretical guidance for the rational design of sulfide‐based anode materials.

## 2 Results and Discussion

### 2.1 Preparation and Morphology

**FIGURE 1:**
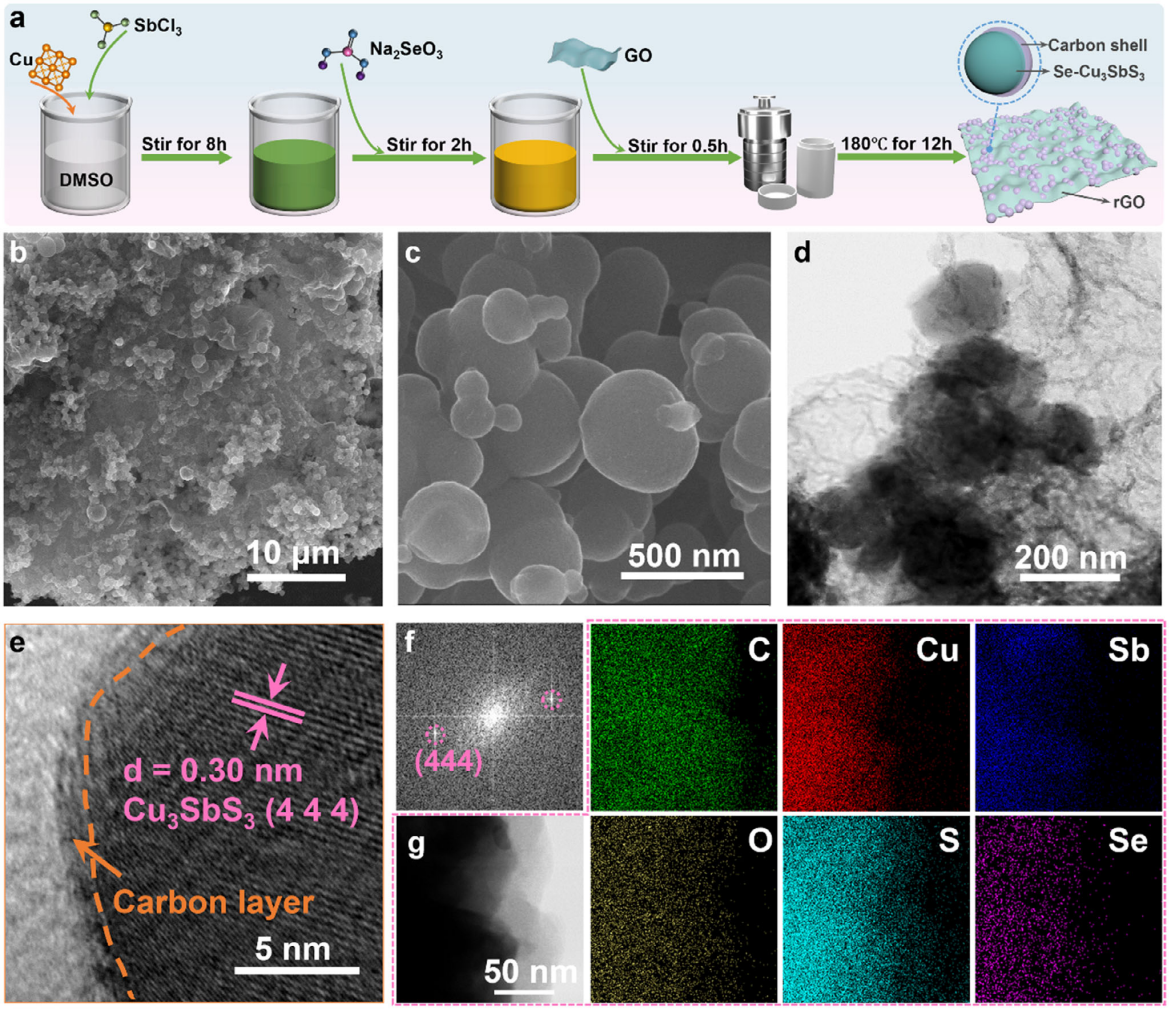
Preparation and morphology of Se‐Cu_3_SbS_3_@rGO. (a) Preparation process of the Se‐Cu_3_SbS_3_@rGO composite. (b, c) SEM images and (d) TEM image showing the morphologies. (e) HRTEM image and (f) selected‐area electron diffraction providing the crystal structures. (g) EDS mapping images displaying the distribution of C, O, Cu, S, Sb, and Se elements.

Se‐Cu_3_SbS_3_@rGO was synthesized via a simple hydrothermal method, as illustrated in Figure 1a. In this process, Cu and SbCl_3_ were first dissolved and thoroughly stirred in dimethylsulfoxide (DMSO) to form a homogeneous green solution, into which sodium selenite was added as the doping source. Subsequently, a certain amount of GO dispersion was introduced as a substrate to support the resulting product. During the hydrothermal treatment, DMSO acts as sulfide sources [28]. After washing and drying, uniformly dispersed nanospheres anchored on the surface of rGO nanosheets were obtained (Figure 1b). These nanospheres, with diameters of approximately 200–300 nm, exhibited relatively smooth surfaces and partial interparticle fusion (Figure 1c). Cu_3_SbS_3_@rGO displayed a similar morphology, while neat rGO only shows wrinkled and stacked nanosheets (Figure S1). TEM analysis (Figure 1d) further confirmed the presence of wrinkled rGO layers with the adhered solid nanospheres. Within the nanospheres, both amorphous carbon layers and obvious lattice fringes were observed (Figure 1e). The lattice spacing of ∼0.30 nm corresponds to the (444) planes of Cu_3_SbS_3_, which is consistent with the selected area electron diffraction (SAED) pattern shown in Figure 1f. These results confirm that the nanospheres are primarily composed of Cu_3_SbS_3_ and amorphous carbon, the latter likely derived from the decomposition of DMSO. High‐resolution TEM images in Figure S2a,b further reveal a distorted crystalline structure with obvious defects in the Se‐doped samples, in contrast to the well‐ordered lattice observed in pure Cu_3_SbS_3_ (Figure S2d). These structural differences highlight the substantial influence of Se‐vacancies doping on the crystal lattice. Elemental mapping within the nanosphere region reveals a homogeneous distribution of C, Cu, Sb, and S (Figure 1g). In addition, uniform signals of O and Se were detected. The presence of oxygen may originate from oxygen‐containing groups in the amorphous carbon, while the homogeneous distribution of Se provides clear evidence of its successful incorporation into the Cu_3_SbS_3_.

### 2.2 Structural Characterization

**FIGURE 2:**
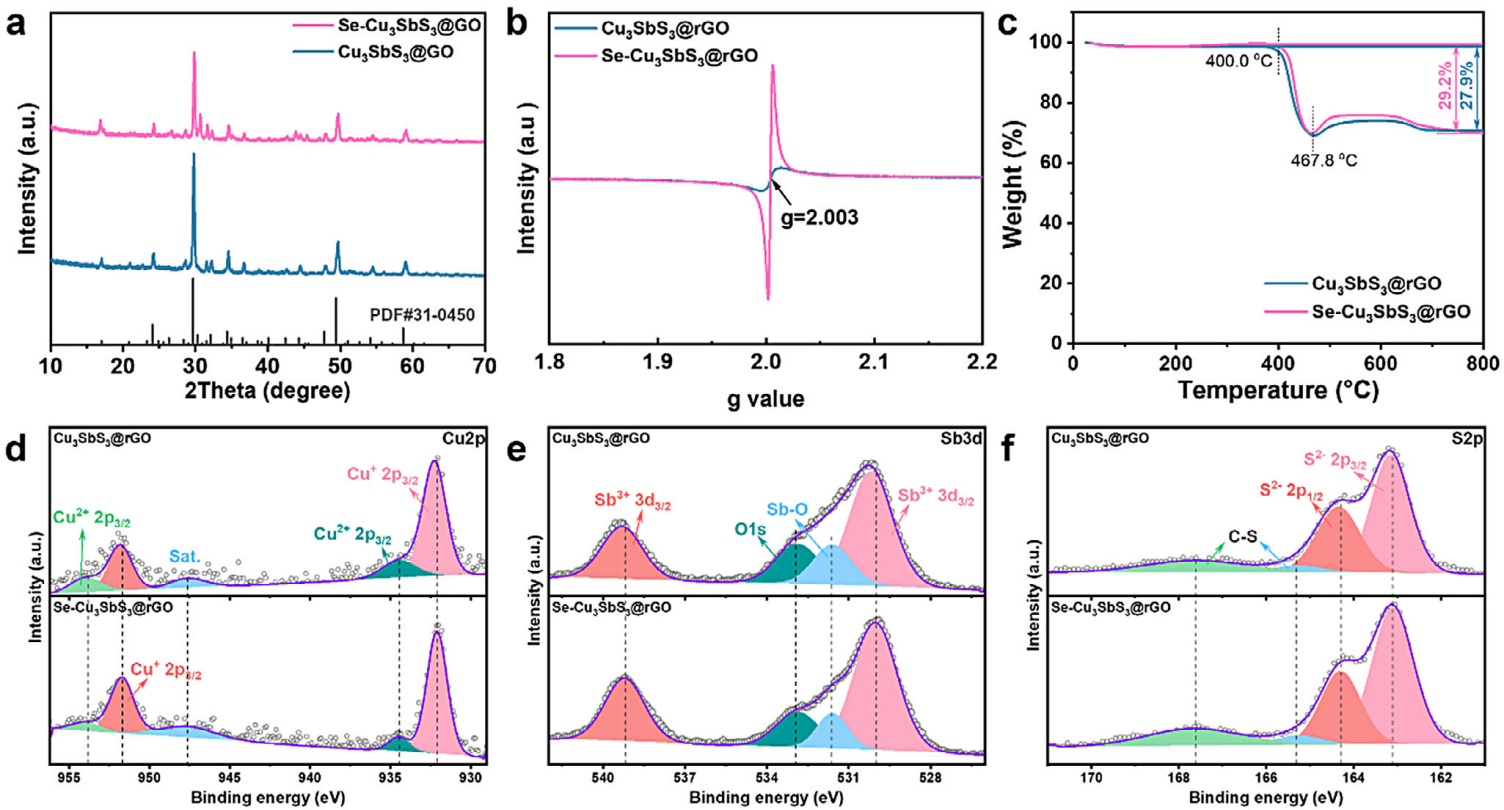
Structural characterizations of the Se‐Cu_3_SbS_3_@rGO composite. (a) XRD patterns, (b) EPR spectra, (c) TG curves of the Se‐Cu_3_SbS_3_@rGO, and Cu_3_SbS_3_@rGO samples. High‐solution XPS spectra of the (d) Cu 2p, (e) Sb 3d, and (f) S 2p.

Further compositional and chemical state characterization was conducted using various spectroscopic techniques. As shown in Figure 2a, both Se‐Cu_3_SbS_3_@rGO and the reference sample Cu_3_SbS_3_@rGO exhibited identical XRD patterns matching Cu_3_SbS_3_ (PDF#21‐0450), consistent with the TEM results and confirming the phase purity. Figure 2b presents the EPR spectra, where the Se‐doped sample shows a significantly enhanced signal at g = 2.003. This pronounced peak is indicative of a large number of sulfur vacancies (*V*_s_) [29, 30]. This phenomenon is likely attributed to the difference in ionic radii and electronegativity between Se and S. The larger Se atoms induce lattice distortion and concomitant S vacancies, which further exacerbate the lattice distortion to modulate the electronic properties of Cu_3_SbS_3_. Figure 2c shows the thermogravimetric analysis (TGA) curves of the two samples measured in air. Both samples exhibit noticeable weight loss beginning at around 400°C, which is attributed to the oxidation of rGO and amorphous carbon into CO_2_ [31]. The observed mass change starts from 467.8 °C corresponds to the oxidative decomposition of Cu_3_SbS_3_ [31, 32, 33]. The final mass loss at 800 °C were 29.2% for Se‐Cu_3_SbS_3_@rGO and 27.9% for Cu_3_SbS_3_@rGO, suggesting a carbon content of 25.8% and 24.7%, respectively. It also evidences the closed Cu_3_SbS_3_ content in the two samples.

X‐ray photoelectron spectroscopy (XPS) was employed to investigate the chemical states of the elements in Se‐Cu_3_SbS_3_@rGO. As shown in Figure S3a, the C 1s spectra of both samples exhibit four distinct peaks located at 284.8, 285.6, 286.7, and 288.8 eV, which correspond to C─C/C═C, C─S, C─O, and C═O bonds, respectively [6, 34]. The presence of the C─S peak indicates that a small portion of sulfur existed on the rGO surface or in amorphous carbon. The Cu 2p spectra of both samples (Figure 2d) display a pair of strong peaks assigned to Cu^+^ species from Cu_3_SbS_3_, along with a pair of weaker peaks corresponding to a small amount of surface‐oxidized Cu^2+^ [26]. Notably, the Cu^+^ binding energies in Se‐Cu_3_SbS_3_@rGO are slightly shifted to lower values. This shift is likely due to the combined effect of Se doping and S vacancies, which induce partial electron localization on Cu atoms and weaken the Cu─S bonding strength [17]. A similar trend is observed in the Sb 3d spectra (Figure 2e), which include a peak corresponding to O 1s, a peak related to Sb─O species, and a pair of peaks corresponding to Sb^3+^ [31, 35]. Compared with Cu_3_SbS_3_@rGO, the Sb^3+^ binding energies in Se‐Cu_3_SbS_3_@rGO are also reduced, indicating a weakening of the Sb─S bonds as a result of the lattice distortion. Correspondingly, the weakening of both Cu─S and Sb─S bonds influences the chemical environment and bonding states of sulfur. As shown in Figure 2f, both samples exhibit a pair of peaks attributed to S^2−^ in Cu_3_SbS_3_ and a pair of minor peaks corresponding to S─C bond [36]. It is evident that the S^2−^ peaks in Cu_3_SbS_3_@rGO are located at higher binding energies compared to those in Se‐Cu_3_SbS_3_@rGO. Figure S3b shows the Se 3d XPS spectra, where two distinct peaks corresponding to Se 3d_3/2_ and Se 3d_5/2_ are clearly observed in Se‐Cu_3_SbS_3_@rGO [9], but are absent in the undoped Cu_3_SbS_3_@rGO sample. This further confirms the successful incorporation of Se in the Se‐Cu_3_SbS_3_@rGO composite. XPS results revealed a Se concentration of 0.93% in the Se‐Cu_3_SbS_3_@rGO composite, with a corresponding Se/(S+Se) atomic ratio of 0.0585.

### 2.3 Sodium‐Ion Storage Performance

**FIGURE 3:**
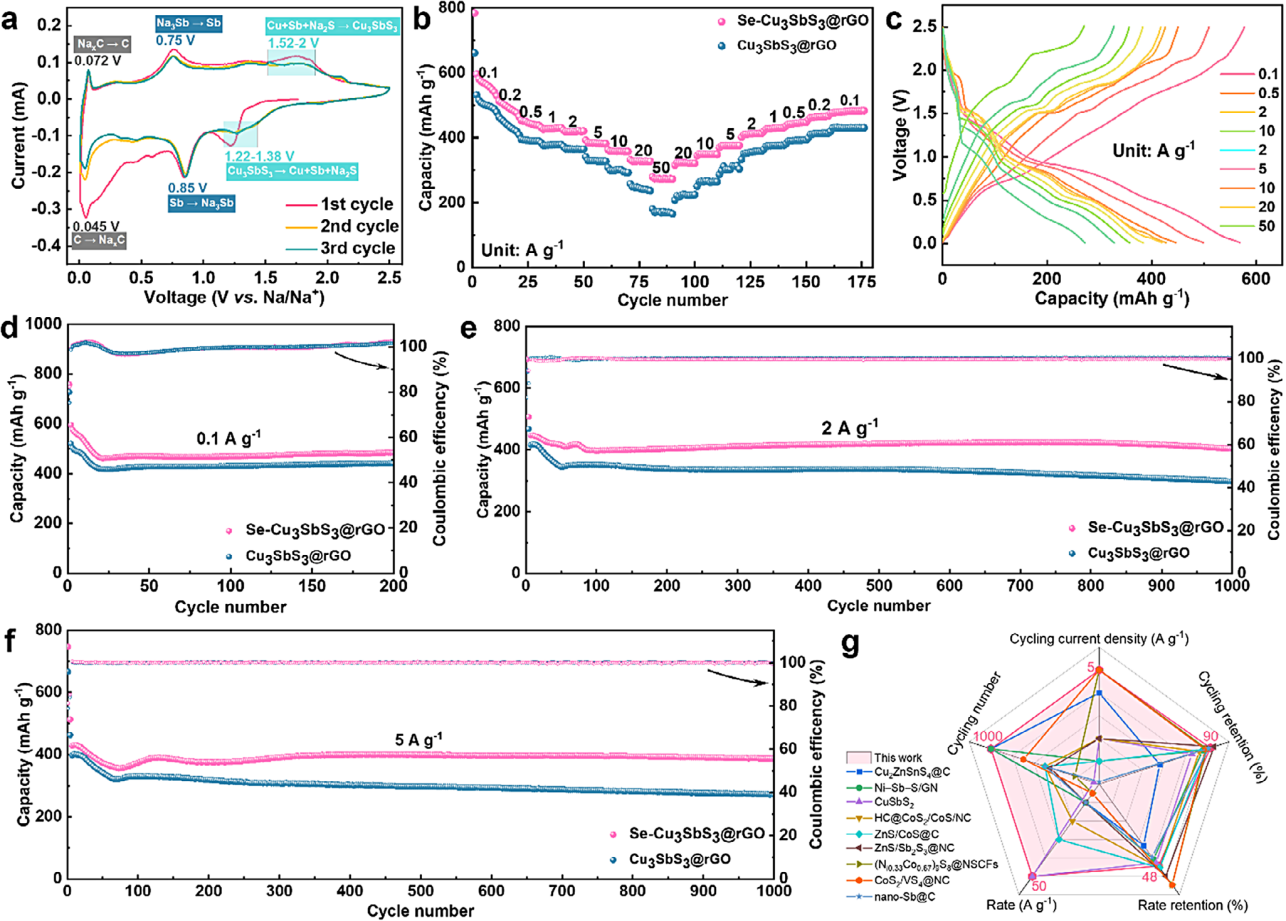
Sodium storage performance of the Se‐Cu_3_SbS_3_@rGO anodes. (a) The first three CV curves of Se‐Cu_3_SbS_3_@rGO. (b) Rate performance of Se‐Cu_3_SbS_3_@rGO and Cu_3_SbS_3_@rGO samples from 0.1 to 50 A g^−1^. (c) Charge–discharge profiles of Se‐Cu_3_SbS_3_@rGO at different current densities. Cycling performances of Se‐Cu_3_SbS_3_@rGO and Cu_3_SbS_3_@rGO anodes at (d) 0.1 A g^−1^, (e) 2 A g^−1^, and (f) 5 A g^−1^. (g) Comparison of electrochemical performance between Se‐Cu_3_SbS_3_@rGO and other state‐of‐art anodes.

Electrochemical tests were conducted to evaluate the sodium storage performance of the two samples. They were first tested in half cells with a mass loading of about 1–1.2 mg cm^−2^. Figure 3a shows the first three cyclic voltammetry (CV) curves of Se‐Cu_3_SbS_3_@rGO. During the initial discharge process, three prominent reduction peaks are observed. The peak at 1.22 V corresponds to the conversion reaction producing Cu, Sb, and Na_2_S [12, 37]. In subsequent cycles, this peak evolves into a broad signal in the 1.22–1.38 V range, which may be attributed to nano‐crystallization caused by the structural reconstruction. The reduction peak at approximately 0.85 V is assigned to the alloying reaction between Sb and Na^+^ to form Na_3_Sb [38]. The final reduction peak near 0.045 V can be attributed to the sodium storage process in the carbon matrix, forming Na_x_C [39, 40]. During the charging process, sharp oxidation peaks are observed at 0.072 and 0.75 V, corresponding to the desodiation process from Na_x_C and Na_3_Sb, respectively. A broad peak in the 1.5–2.0 V range is associated with the reformation of Cu_3_SbS_3_. In the following charge–discharge cycles, the redox peaks exhibit excellent overlap, indicating a good reversibility of Se‐Cu_3_SbS_3_@rGO. This observation is further supported by the galvanostatic charge–discharge curves shown in Figure S4, where both samples display similar behavior. In addition, Se‐Cu_3_SbS_3_@rGO displayed an initial capacity of 758.71 mAh g^−1^ with an initial coulombic efficiency (ICE) of 82.5%. The capacity loss should be attributed to the irreversible capacity loss caused by SEI formation, ion adsorption at defects and loss of active materials.

Se‐Cu_3_SbS_3_@rGO composites with varying Se contents were fabricated to evaluate the effect of doping concentration. ICP‐OES was further employed to determine the bulk Se contents of the Se‐Cu_3_SbS_3_@rGO samples (Figure S5a). The measured Se concentrations are 0.49%, 0.90%, and 1.25% for the samples synthesized with 0.5, 1.0, and 1.5 mmol Na_2_SeO_3_, respectively, confirming that the bulk compositions are consistent with the intended doping levels. As shown in Figure S5b, increasing the Se‐doping level enhances the cycling stability, confirming the critical role of Se incorporation in improving the electrochemical performance. However, when the Se content increases to 1.25%, a more stable yet lower capacity is observed, which may result from excessive sulfur loss at this higher doping level. Figure 3b presents the rate performance at different current densities. The Se‐Cu_3_SbS_3_@rGO electrode delivered reversible capacities of 569.1 (5th), 498.7 (15th), 446.1 (25th), 427.4 (35th), 419.3 (45th), 383.6 (55th), 357.5 (65th), and 328.4 (75th) mAh g^−1^ at current densities of 0.1, 0.2, 0.5, 1, 2, 5, 10, and 20 A g^−1^, respectively, which are all significantly higher than those of the undoped Cu_3_SbS_3_@rGO. Even under an ultrahigh current density of 50 A g^−1^, the Se‐doped sample still retained a high capacity of 273.6 mAh g^−1^ (85th), corresponding to a 48% capacity retention relative to its initial value at 0.1 A g^−^^1^. In contrast, Cu_3_SbS_3_@rGO only maintained a low capacity of 168 mAh g^−1^ and a retention of 33% under the same conditions. When the current density was gradually returned to 0.1 A g^−1^, the capacity of Se‐Cu_3_SbS_3_@rGO recovered to as high as 482.8 mAh g^−1^, while that of Cu_3_SbS_3_@rGO only recovered to 430.8 mAh g^−1^, further confirming its inferior performance. Figure 3c and Figure S6 show the charge–discharge profiles of the two anodes under various current densities. It can be observed that Se‐Cu_3_SbS_3_@rGO exhibits lower voltage polarization and smaller capacity fading as the current rates. These results solidly validate the effectiveness of Se─V_s_ incorporation on promoting the reaction kinetics. Performance of pure rGO anodes was also collected to assess the capacity contribution from carbon (Figure S7). Combined with the TGA results, the carbon‐contributed capacity was calculated to be 6.1% at 0.1 A g^−1^ and 7.2% at 50 A g^−1^, confirming the dominant contribution from Se‐doped Cu_3_SbS_3_.

To further evaluate the effect of Se─V_s_ doping on electrochemical performance, cycling stability tests were carried out at different current densities. Figure 3d shows the capacity evolution over 200 cycles at a current density of 0.1 A g^−1^. Following the initial capacity drop during the early 20 cycles, both electrodes exhibited stable cycling behavior. To clarify the origin of Coulombic efficiencies exceeding 100%, we examined the charge–discharge curves of the affected cycles (Figure S8a), which show a new redox pair at ∼1.95 V (discharge) and ∼2.1 V (charge), associated with sulfur conversion [41]. Disassembly after 30 cycles revealed a light‐yellowed separator, and UV–vis of the cycled separator shows a clear absorption peak corresponding to Na_2_S_x_, confirming the formation of soluble sodium polysulfides (Figure S8b). During cycling, these polysulfides shuttle between electrodes, undergoing reversible redox reactions and contributing to extra charge transfer, which explains both the CE > 100% and the irregular capacity evolution in early cycles. Such a behavior has also been found in other sulfides‐based anodes [42, 43, 44]. However, the polysulfide shuttling effect in the Se‐Cu_3_bS_3_@rGO anode appears to be limited to a minor extent. The shuttle‐related capacity accounts for a lower ratio of 3.1% compared with 4.2% for Cu_3_SbS_3_@rGO (Figure S8c), which is consistent with its excellent long‐term cycling stability. After 200 cycles, Se‐Cu_3_SbS_3_@rGO and Cu_3_SbS_3_@rGO delivered reversible capacities of 483.6 and 441.5 mAh g^−1^, respectively. Long‐term cycling tests were further performed under high current densities. At 2 A g^−1^ (Figure 3e), Se‐Cu_3_SbS_3_@rGO maintained a high capacity of 403.8 mAh g^−1^ after 1000 cycles, corresponding to 90.2% capacity retention, which is significantly higher than that of the reference sample (298.5 mAh g^−1^, 72% retention). At an even higher current density of 5 A g^−1^ (Figure 3f), Se‐Cu_3_SbS_3_@rGO still retained 387.4 mAh g^−1^ after 1000 cycles (90.0% retention), whereas Cu_3_SbS_3_@rGO dropped to 270.7 mAh g^−1^ (68.6% retention). The rapid capacity fading in the initial cycles (Figure 3d–f) is mainly attributed to the formation and stabilization of the SEI, structural degradation induced by volume expansion, and the loss of few soluble polysulfides formed in early cycles. As cycling proceeds, the SEI stabilizes and the electrode structure adapts, reducing these capacity loss pathways and resulting in more stable performance. The electrochemical performance of the Se‐Cu_3_SbS_3_@rGO anode was further evaluated under a high areal loading of ∼6 mg cm^−2^ (Figure S9). Even at this loading, the anode delivered a high capacity of 484.9 mAh g^−1^ at 0.1 A g^−1^ and retained 329.1 mAh g^−1^ at 5 A g^−1^, demonstrating excellent rate capability. It also exhibited remarkable cycling stability, maintaining 447.0 mAh g^−1^ (84.5%) after 100 cycles at 0.1 A g^−1^ and 376.1 mAh g^−1^ (87.1%) after 1000 cycles at 2 A g^−1^. Such a result once again confirming the critical role of Se─V_s_ doping on the structure and sodium‐storage performance. Even compared with recently reported state‐of‐the‐art sulfide‐based anodes (Figure 3g; Table S1), this Se‐Cu_3_SbS_3_@rGO clearly showed its rate capability and cycling stability [12, 36, 38, 45, 46, 47, 48, 49].

### 2.4 Reaction Kinetics

**FIGURE 4:**
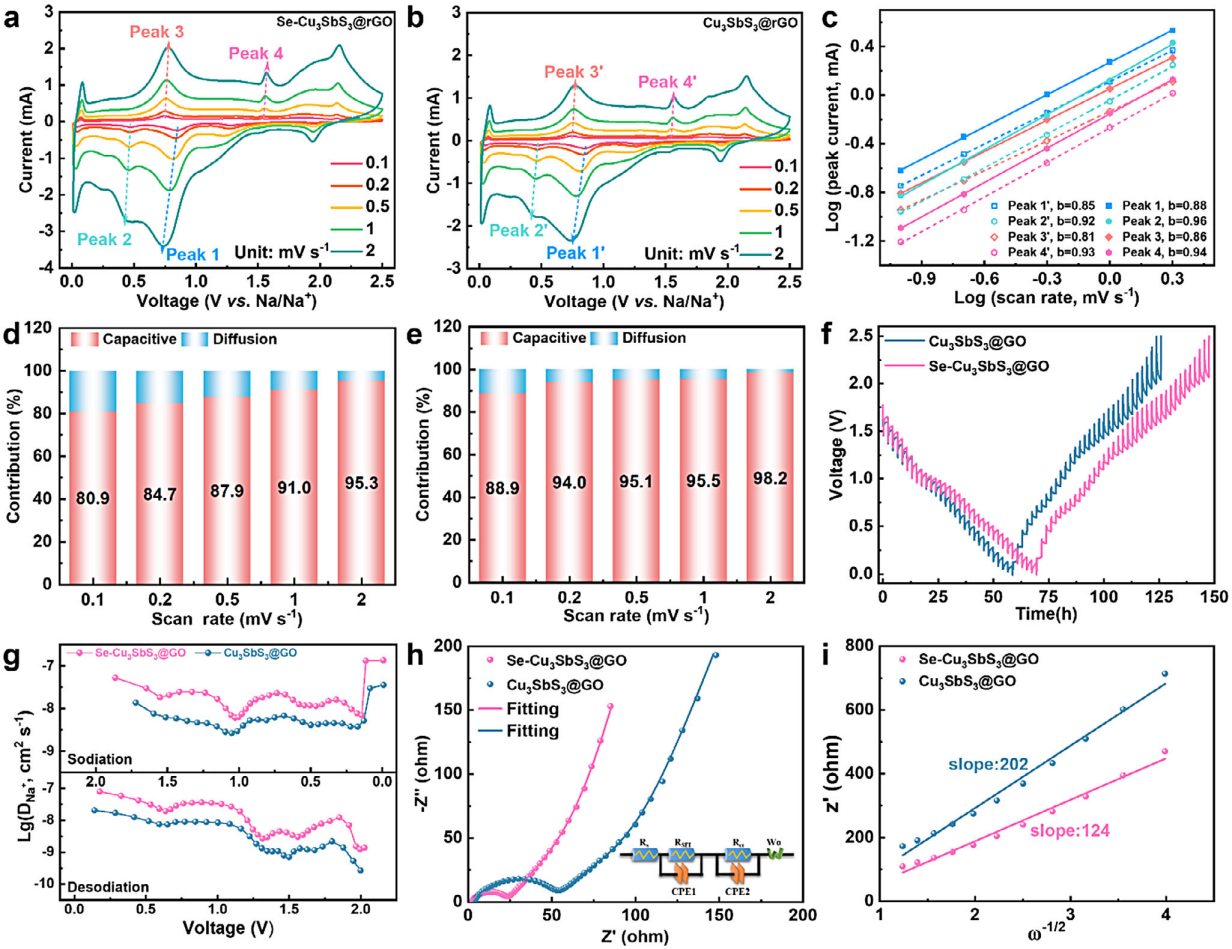
Electrochemical reaction kinetics of the Se‐Cu_3_SbS_3_@rGO. CV curves at different scan rates of (a) Se‐Cu_3_SbS_3_@rGO and (b) Cu_3_SbS_3_@rGO. (c) Fitted b values of the marked peaks in (a) and (b). Capacitive contribution at different rates of (d) Se‐Cu_3_SbS_3_@rGO and (e) Cu_3_SbS_3_@rGO. (f) GITT profiles and (g) the corresponding Na^+^ diffusion coefficient. (h) Comparison of the EIS results. (i) Plots and fitted slopes between ω^−1/2^ and Z′.

To further validate the role of Se─V_s_ doping, electrochemical reaction kinetics were investigated by multi‐CV scans, GITT, and EIS. Figure 4a,b presents the CV curves of Se‐Cu_3_SbS_3_@rGO and Cu_3_SbS_3_@rGO at scan rates ranging from 0.1 to 2 mV s^−1^, exhibiting similar redox features. Notably, the reduction peaks (peak 1 and peak 2) of Se‐Cu_3_SbS_3_@rGO are located at slightly higher potentials than those of the control sample at all scan rates, indicating a reduced electrochemical polarization. More importantly, the potential shifts with increasing scan rate are approximately 0.1 V for peak 1 and 0.02 V for peak 2 in Se‐Cu_3_SbS_3_@rGO, which are smaller than those observed for Cu_3_SbS_3_@rGO (∼0.12 and ∼0.03 V, respectively). This behavior suggests lower polarization enhancement and improved rate capability for Se‐Cu_3_SbS_3_@rGO. Based on Equation S1, the b values were extracted from the logarithmic relationship between the peak currents and scan rates (Figure 4c). Obviously, all b values of Se‐Cu_3_SbS_3_@rGO are closer to 1 and consistently higher than those of the undoped sample, illustrating its capacitive‐dominated electrochemical process [50, 51]. According to Equation S2, the capacity contributions from capacitive and diffusion process were quantitatively acquired at different scan rates (Figure 4d,e) [52, 53]. Se‐Cu_3_SbS_3_@rGO exhibited a higher capacitive contribution at all scan rates. For example, it accounts for 95.1% of the total current response in Se‐Cu_3_SbS_3_@rGO at a scan rate of 0.5 mV s^−1^ (Figure S10), which is significantly higher than that of the reference Cu_3_SbS_3_@rGO electrode (87.9%). These findings highlight the capacitive‐dominated reaction and enhanced reaction kinetics imparted by the co‐doping of Se and sulfur vacancies. CV measurements at higher sweep rates (Figure S11) also reveal good linearity between log(peak current) and log(scan rate), yielding high b values that verify the dominant capacitive contribution in Se‐Cu_3_SbS_3_@rGO at high rates. To further evaluate sodium‐ion transport behavior, GITT measurements were conducted as shown in Figure 4f. The Na^+^ diffusion coefficients (*D*_Na+_) during charge/discharge process were calculated using Equation S3 [46, 53]. Both samples exhibited similar trends in *D*_Na+_ evolution (Figure 4g), suggesting comparable reaction pathways. However, Se‐Cu_3_SbS_3_@rGO consistently demonstrated significantly higher *D*_Na+_ values throughout the entire process, which can be attributed to the accelerated Na^+^ transport enabled by Se─V_s_ doping. Electrochemical impedance spectroscopy (EIS) was also performed to probe interfacial and bulk transport properties (Figure 4h). The Nyquist plots exhibit a semicircle in the high‐frequency region involving the SEI resistance and charge transfer resistance at the electrode–electrolyte interface, and a sloped line in the low‐frequency region that is associated with Warburg diffusion [54]. The smaller semicircle of Se‐Cu_3_SbS_3_@rGO indicates its rapid interfacial process. According to Equation S4, the Warburg coefficient (σ) was obtained by fitting the linear portion of Z′ versus ω^−1/2^ [26, 47]. The σ values were calculated to be 124 for Se‐Cu_3_SbS_3_@rGO and 202 for Cu_3_SbS_3_@rGO, respectively. Based on the ion diffusion rate relationship (Equation S5), a smaller σ represents faster ion diffusion, which agrees well with the GITT results [50]. Overall, these results provide compelling evidence that Se─V_s_ doping significantly improves the ion diffusion rate and the reaction kinetics of Se‐Cu_3_SbS_3_@rGO electrodes, thereby contributing to their outstanding sodium storage performance.

### 2.5 DFT Calculation

**FIGURE 5:**
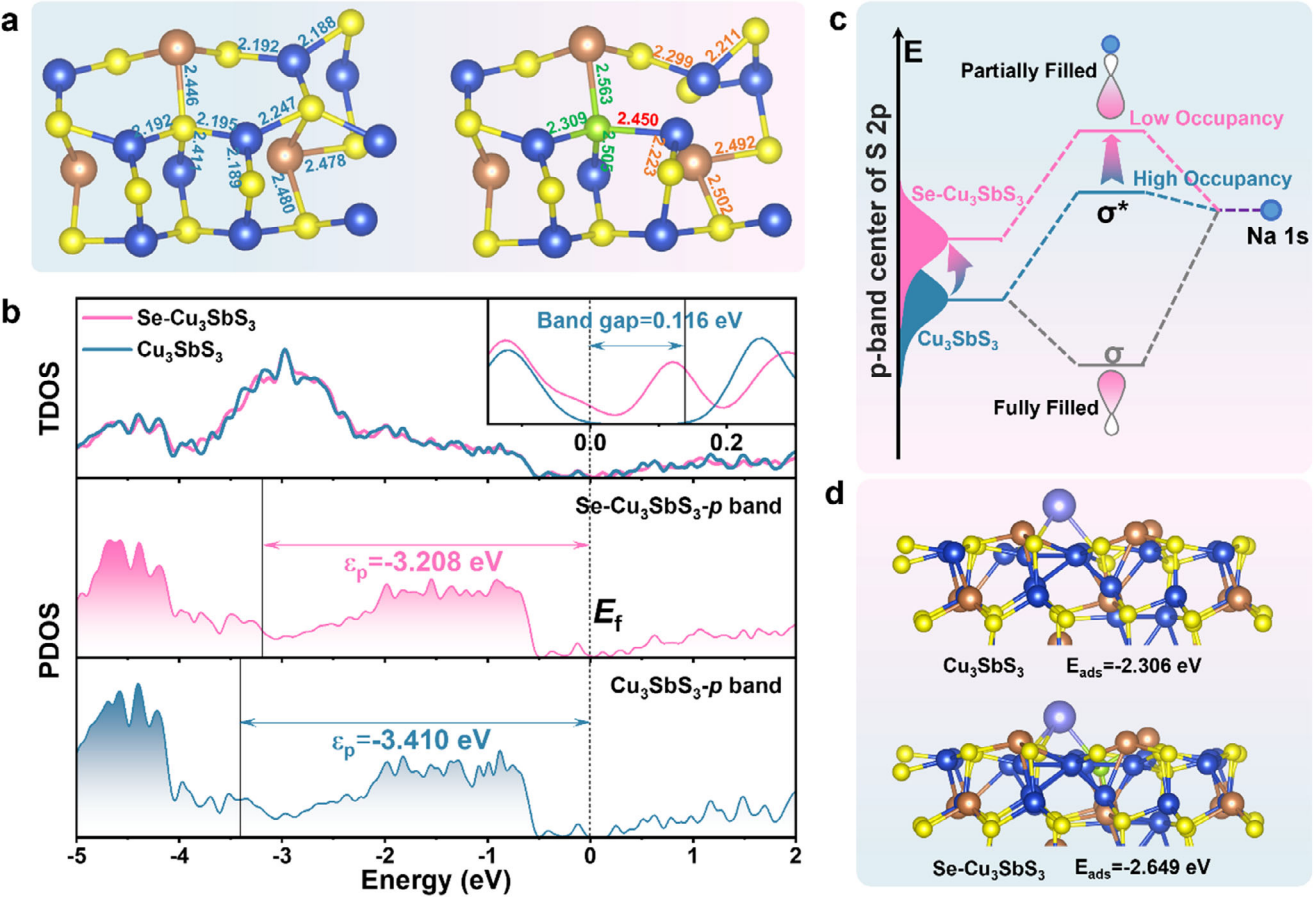
Density functional theory (DFT) calculations. (a) The crystal structures of Se‐Cu_3_SbS_3_ and Cu_3_SbS_3_. (b) The density of states (DOS) and p‐band centers of Se‐Cu_3_SbS_3_ and Cu_3_SbS_3_. (c) Schematic illustrating the mechanism of modulating the p‐band center of Cu_3_SbS_3_ for enhanced Na^+^ adsorption. (d) The calculated adsorption energy of Na^+^ on the surfaces of Se‐Cu_3_SbS_3_ and Cu_3_SbS_3_.

To further investigate the underlying mechanism, DFT calculations were conducted to analyze the crystal and electronic structures of Se‐Cu_3_SbS_3_. Since rGO substrate was used in both samples and has minimal impact on their morphologies, the calculation models were simplified to focus on the intrinsic effects of Se doping and sulfur vacancies. As shown in Figure 5a, comparison of the lattice structures of Se‐Cu_3_SbS_3_ and pristine Cu_3_SbS_3_ reveals that the bonds surrounding the Se atoms and sulfur vacancies were all elongated. In particular, the bond length affected by both Se doping and S vacancies increases from 2.195 to 2.45 Å, indicating that such dual doping induces pronounced lattice distortion. Differential charge density distributions in Figure S12 further reveal a reduced charge distribution at the metal–nonmetal bonds near Se doping sites and S vacancies in Se‐doped Cu_3_SbS_3_. This weakening of bonding is consistent with the observed bond elongation and is favorable for elevating the p‐band center [24]. The density of states (DOS) is presented in Figure 5b. While the total DOS profiles of the two structures are similar, a magnified view around the Fermi level shows that the small bandgap present in pristine Cu_3_SbS_3_ is eliminated. The introduction of Se and vacancies should be the reason that increases the concentration of charge carriers, thereby enhancing the electronic conductivity [55]. According to the PDOS analysis, the p‐band center of Se‐Cu_3_SbS_3_ is located at −3.208 eV, higher than that of Cu_3_SbS_3_ (−3.410 eV), indicating that the elongated bonds modulate charge redistribution and shift the p‐band upward. As illustrated in Figure 5c, the elevation of the p‐band center is able to reduce the occupancy of Na─S antibonding σ* orbitals, thereby strengthening the interaction between Na^+^ and S in sulfides [17, 21]. Consequently, Se‐Cu_3_SbS_3_ exhibits an improved Na^+^ affinity with an adsorption energy of −2.649 eV, compared to −2.306 eV for the undoped sample. In summary, the introduction of larger Se atoms into Cu_3_SbS_3_ induces the simultaneous formation of accompanying sulfur vacancies. The synergistic effect of Se doping and S vacancies leads to significant lattice distortion, characterized by elongated metal–nonmetal bonds. The resulting weakened hybridization contributes to the elevation of the p‐band center, thus enhances Na^+^ adsorption to promote the reaction kinetics and overall electrochemical performance.

Overall, the core mechanism of this work lies in the incorporation of Se atoms with a larger size and weak electronegativity, which induces local lattice stress and the generation of accompanied sulfur vacancies. These vacancies further exacerbate lattice distortion, ultimately enabling effective modulation of electronic properties. Similar performance enhancements have been also observed in other systems, such as CuS for Zn^2+^ storage [56], In‐MoS_2_ for sulfur redox [57] and ZnCdS for photocatalytic activity [58], suggesting the universality and effective of engineering S vacancies or p‐band center. However, a few of them are concerned about the lattice distortion and its influence on the electronic properties. In this regard, this study highlights a broadly applicable strategy for the rational design of active materials for Na^+^ storage electrodes. By introducing dopant atoms with different sizes and electronegativities to induce accompanying vacancies, a comparable doping–vacancy synergistic effect can be realized to modulate the lattice distortion and electronic properties of various active materials, such as oxides, sulfides, selenide et al.

### 2.6 Reaction Mechanism and Full Cell

**FIGURE 6:**
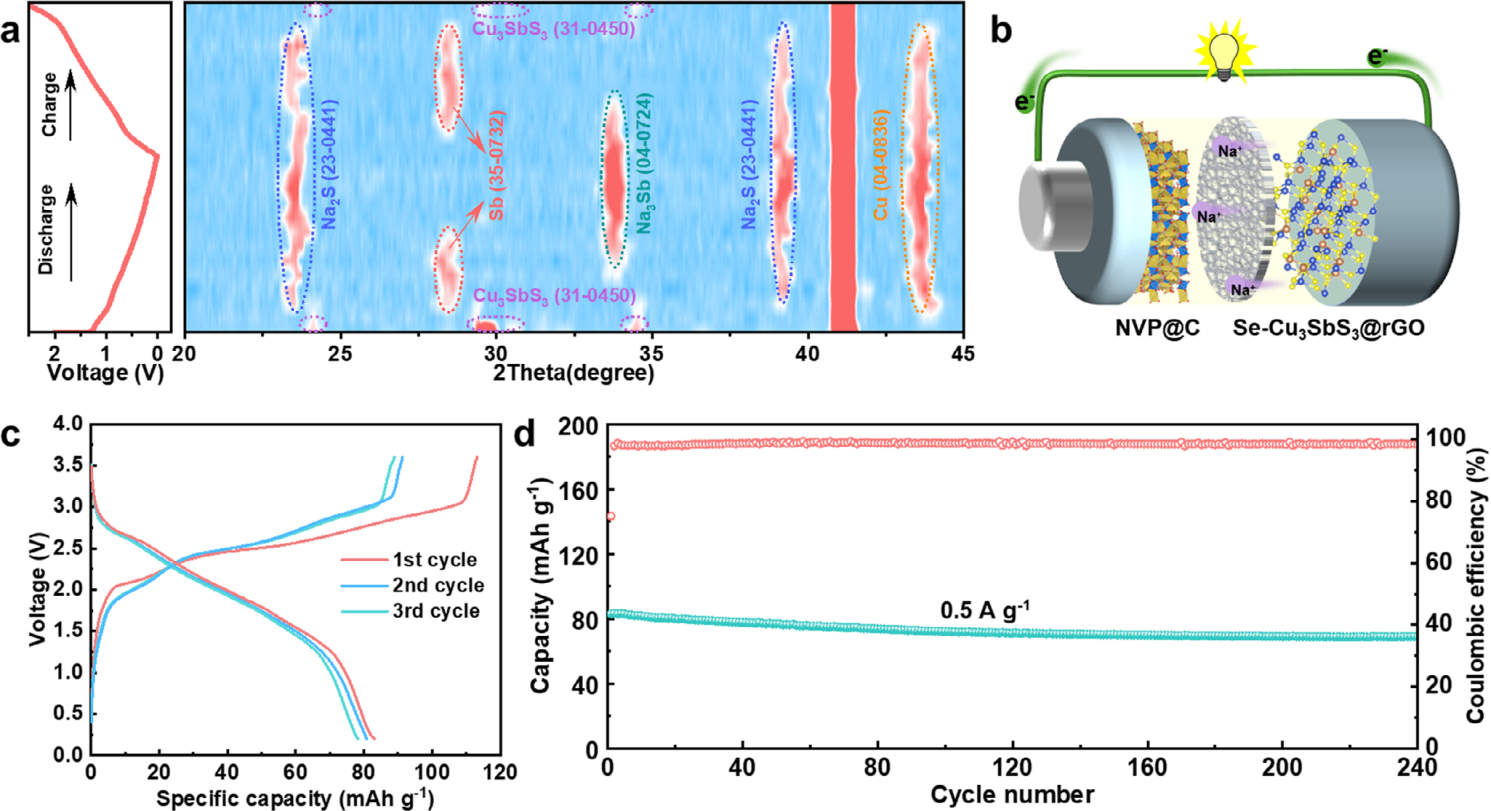
Reaction process and practical verification of Se‐Cu_3_SbS_3_@rGO. (a) In situ XRD patterns of Se‐Cu_3_SbS_3_@rGO during charge‐discharge process. (b) Configuration of the NVP@C∥Se‐Cu_3_SbS_3_@rGO full cell. (c) The first three charge–discharge profiles and (d) the cycling performance of the NVP@C∥Se‐Cu_3_SbS_3_@rGO full cell.

In situ XRD was employed to investigate the reaction mechanism and phase evolution of Se‐Cu_3_SbS_3_@rGO during electrochemical process (Figure 6a; Figure S13). At the beginning of discharge, three distinct diffraction peaks corresponding to the (440), (444), and (800) planes of Cu_3_SbS_3_ (PDF#31‐0450) were clearly observed. As the reaction progressed, new peaks emerged at 23.5° and 38.9°, which could be indexed to the (111) and (220) planes of Na_2_S (PDF#23‐0441), respectively. Additionally, peaks appeared at 28.7° and 43.3°, corresponding to the (012) plane of metallic Sb (PDF#35‐0732) and the (111) plane of metallic Cu (PDF#04‐0836), respectively. The simultaneous appearance of Na_2_S, Sb, and Cu signals confirmed the occurrence of a conversion reaction. Subsequently, the intensity of the Sb peak gradually decreased, while a new peak emerged near 34°, attributed to Na_3_Sb (PDF#04‐0836), indicating the progression of a sodiation alloying reaction. During the desodiation process, these peak changes occurred in reverse order: the Na_3_Sb peak diminished first, followed by the reappearance of the Sb peak, and finally the disappearance of the Na_2_S, Sb, and Cu peaks accompanied by the recovery of the Cu_3_SbS_3_ peaks. Notably, the reformed Cu_3_SbS_3_ peaks exhibited reduced intensity, likely due to the decreased crystallinity caused by the conversion‐induced reconstruction [17, 59]. These findings are consistent with the CV analysis and further validate the good reversibility of Se‐Cu_3_SbS_3_@rGO. Based on the above results, the sodiation/desodiation process of Se‐Cu_3_SbS_3_@rGO can be described by the following reactions:

Discharging process:

Cu3SbS3+6Na++6e−→3Cu+Sb+3Na2S

Sb+3Na++3e−→Na3Sb

Charging process:

Na3Sb→Sb+3Na++3e−

3Cu+Sb+3Na2S→Cu3SbS3+6Na++6e−

To elucidate the morphology evolution and SEI structure, TEM, SEM, and XPS analyses were conducted (Figures S14–S16). TEM images (Figure S14) of the Se‐Cu_3_SbS_3_@rGO electrode after 50 and 1000 cycles both reveal the (444) planes of Cu_3_SbS_3_ with a spacing of 0.30 nm, confirming the preservation of the active phase even after long‐term cycling. The observed lattice discontinuity and reduced crystallinity should be resulted from the grain refinement during structural reconstruction in the early stage of cycling, consistent with in situ XRD, which explains the initial capacity fluctuations. SEM observations (Figure S15) after 1000 cycles at 5 A g^−1^ show that the pristine electrode suffers from severe cracks, surface roughness, and pulverized particles, whereas the Se‐doped electrode retains a uniform morphology with well‐preserved nanoparticles. Furthermore, XPS analysis (Figure S16) reveals a higher NaF content and fewer organic species in the SEI of the Se‐Cu_3_SbS_3_@rGO electrode, consistent with the SEM results. In contrast, the pristine sample undergoes structural degradation that promotes more side reactions and organic accumulation [60]. Collectively, these results demonstrate the excellent structural stability of the Se‐Cu_3_SbS_3_@rGO electrode, highlighting the role of enhanced kinetics in homogenizing volume changes and stabilizing the electrode during cycling.

To assess the practical applicability of Se‐Cu_3_SbS_3_@rGO, a full cell was assembled using Na_3_V_2_(PO_4_)_3_@C (NVP@C) as the cathode material (Figure 6b). The electrochemical performance of NVP@C cathode is shown in Figure S17, displaying a stable voltage plateau around 3.4 V. The cathode delivered specific capacities of 103.3, 98.1, 95.5, and 93.5 mAh g^−1^ at 0.1, 0.2, 0.5, and 1 A g^−1^, respectively. After 100 cycles at 1 A g^−1^, the NVP@C electrode retained a capacity of 90 mAh g^−1^, indicating an excellent cycling stability. A P/N ratio of 1.1 was employed to fully utilize the performance of Se‐Cu_3_SbS_3_@rGO anodes, which led to similar capacity fluctuations to those observed in the half cells. Based on the mass of the NVP@C cathode, the assembled NVP@C∥Se‐Cu_3_SbS_3_@rGO full cell delivered an initial discharge capacity of 80.8 mAh g^−1^ at 0.5 A g^−1^ (Figure 6c). Even after 240 cycles at 0.5 A g^−1^, the full cell delivers a high capacity of 68.8 mAh g^−1^ (Figure 6d), corresponding to a capacity of 350.9 mAh g^−1^ for the Se‐Cu_3_SbS_3_@rGO anode. Such outstanding stability, characterized by a high retention ratio of 83%, highlights the significant practical potential of the Se‐Cu_3_SbS_3_@rGO anode. Furthermore, a pouch cell was fabricated and tested at 0.5 A g^−1^ (Figure S18). It could retain 59.4% of its capacity (49.9 mAh g^−1^) after 60 cycles and also able to light a “ZZU” shaped LED bulbs, confirming its robustness and practical application potential.

## 3 Conclusion

In summary, a Se‐doped Cu_3_SbS_3_ nanosphere composite anchored on reduced graphene oxide (Se‐Cu_3_SbS_3_@rGO) was successfully designed and synthesized through a simple hydrothermal method. The introduction of large Se atoms enabled the generation of accompanying S vacancies, which collaborated to induced significant lattice distortion. The bond lengths between metal and nonmetal atoms were increased to weaken their hybridization, and thus raised the sulfur p‐band center. These structural and electronic modifications enhanced the Na^+^ adsorption capability and accelerated the electrochemical reaction kinetics, as supported by DFT calculations, CV kinetics, GITT, and EIS analyses. Benefiting from these structural and electronic advantages, the Se‐Cu_3_SbS_3_@rGO electrode exhibits outstanding sodium‐storage performance, delivering a high reversible capacity of 569.1 mAh g^−1^ at 0.1 A g^−1^, superior rate capability of 273.6 mAh g^−1^ at 50 A g^−1^, and excellent long‐term cycling stability with 387.4 mAh g^−1^ (90.0% retention) after 1000 cycles at 5 A g^−1^. Notably, even at a high areal loading of ∼6 mg cm^−2^, the electrode maintains a high reversible capacity (484.9 mAh g^−1^ at 0.1 A g^−1^), favorable rate performance (329.1 mAh g^−1^ at 5 A g^−1^), and outstanding cycling stability with 87.1% capacity retention after 1000 cycles at 2 A g^−1^, highlighting its strong potential for practical sodium‐ion battery applications. Furthermore, in full‐cell configuration, it preserves 92% of its initial capacity after 150 cycles at 0.5 A g^−1^. This study not only demonstrates the effectiveness of tuning the p‐band center and Na^+^ storage kinetics via the lattice distortion induced by Se─V_s_ doping, but also provides valuable insights into the rational design of high‐performance sulfide‐based anode materials and sodium‐ion batteries.

## Conflicts of Interest

The authors declare no conflicts of interest.

## Supporting information




**Supporting File**: advs75070‐sup‐0001‐SuppMat.docx.

## Data Availability

The data that support the findings of this study are available on request from the corresponding author. The data are not publicly available due to privacy or ethical restrictions.
