# Supplementary material for: Synergistic Se‐Doping and Accompanying S‐Vacancies Drive Lattice Distortion and p‐Band Modulation for Enhanced Sodium Storage in Sulfides
Source: Adv Sci (Weinh). 2026 Apr 2;13(34):e75070. doi: 10.1002/advs.75070 (PMC13285161; doi:10.1002/advs.75070)
Supplement: Supplementary file 1 — Supporting File: advs75070‐sup‐0001‐SuppMat.docx. [file ADVS-13-e75070-s001.docx]

**Supporting Information**

**Synergistic Se-Doping and Accompanying S-Vacancies Drive Lattice Distortion and p-Band Modulation for Enhanced Sodium Storage in Sulfides**

Chunyang Xu, Jinfeng Liu, Biao Ma, Yawen Liang, Dongdong Wang, Chengcheng Miao, Jianhua Zhu, Liu Wang*, Yunfeng Chao*, Xinwei Cui

C. Xu, J. Liu, B. Ma, Y. Liang, J. Zhu, L. Wang, Y. Chao, X. Cui

Henan Institute of Advanced Technology, Zhengzhou University, Zhengzhou 450003, People’s Republic of China

D. Wang

Qinghai Huanghe Hydropower Development Co., Ltd., Xining Qinghai 810000, China

C. Miao

College of Energy Engineering, Huanghuai University, Zhumadian, Henan, 463000, China

Henan Great Power Energy Co. Ltd., Zhumadian, Henan, 463000, China

E-mail: wangliu@zzu.edu.cn (L. Wang), chaoyf@zzu.edu.cn (Y. Chao)

**Experimental Section**

**Synthesis methods.** The synthesis of Se-doped Cu_3_SbS_3_@rGO was carried out by first obtaining a precursor via a solvothermal method, followed by high-temperature calcination under an argon atmosphere. Initially, 1 mmol Cu powder (Aladdin, 99.9%) and 3 mmol SbCl_3_ (Aladdin, 99.9%) were dispersed in 30 mL of dimethyl sulfoxide (DMSO, Sinopharm, 99.5%) under stirring 8 h, forming a uniform bright green solution A. Then, 1 mmol Na_2_SeO_3_ (Aladdin, 99.0%) was dispersed into solution A under stirring for 2 h to form a uniform yellow solution B. Typically, graphene oxide (GO) is prepared by a modified Hummers’ method. Next, 45 mg of GO was added to solution B. The resulting mixture was then transferred into a 100 mL Teflon-lined stainless steel autoclave and maintained at 180 °C for 12 h. After cooling to room temperature, a black precursor was obtained by filtration, washing four times by DMSO and drying at 100 °C for 10 h. Finally, the product was obtained by calcining the precursor at 600 °C for 2 h under an Ar atmosphere at a heating rate of 5 °C/min. The preparation process of the control sample Cu_3_SbS_3_@rGO is similar to that of Se-doped Cu_3_SbS_3_@rGO, except that Na_2_SeO_3_ is not added.

**Material characterization.** The surface morphology of the product was studied by scanning electron microscopy (SEM, ZEISS Gemini 300) with an accelerating voltage of 30 kV. The internal structure and morphology of samples was carried out by transmission electron microscopy (TEM, JEOL-2100F), and high-resolution TEM (HRTEM). The structure and crystal phase of the products were analyzed by X-ray diffraction (XRD, Rigaku Ultima IV). EPS spectra were collected by Bruker EMXplus to analysis the S vacanies. TG was conducted throutgh a Netzsch STA under air at a temperature rate of 5℃ min^-1^. X-ray photoelectron spectrometer (XPS, Thermo Fisher Scientific ESCALAB 250 Xi, USA) was employed to analyze the surface chemical state of the products.

**Electrochemical measurements.** The electrochemical performances of the as-prepared samples were tested using coin-type CR2032 coin cells, which were assembled in an argon glove box (Vigor, LG2400/750TS). The working electrode was obtained by mixing 80 wt% active material, 10 wt% Super P and 10 wt% polyvinylidene fluoride (PVDF) in N-methyl pyrrolidone (NMP) to form a slurry. The homogeneous slurry was then coated on the surface of copper foil and dried overnight under vacuum at 60℃. The loading of the active materials were about 1-1.2 mg cm^-2^. Metallic sodium discs as the counter and reference electrode, and glass fiber (Whatman GF/D) was used as the separator. The electrolyte was 1.0 M of NaPF_6_ dissolved in DME with no additives. The galvanostatic charge-discharge (GCD) tests and galvanostatic intermittent titration technique (GITT) tests were carried out on a Neware testing system (CT4008) with a voltage window from 0.1 to 2.5 V. Cyclic voltammetry (CV) and electrochemical impedance spectroscopy (EIS) measurements were conducted on an electrochemical workstation (Bio-Logic, VSP-3e, France). The full cell was assembled in a similar configuration except using Na_3_V_2_(PO_4_)_3_@C (NVP@C) as the cathodes. A N/P ratio of 1.1 was employed to thoroughly validate the anode performance in full cells, yielding a corresponding mass loading ratio of 5.1:1.”

**Computational Methods.** The Vienna Ab-initio Simulation Package (VASP) was utilized for performing density functional theories (DFT) calculations.^1^ The exchange-correlation effects were modeled using the Perdew-Burke-Ernzerhof (PBE) functional within the framework of Generalized Gradient Approximation (GGA) approach.^2, 3^ The projected augmented wave (PAW) method was employed to describe the core-valence interactions, with a kinetic energy cutoff for the plane wave was set at 400 eV.^4^ Structural optimization was achieved with energy and force convergence criteria set at 1.0×10^-5^ eV and 0.02 eV Å^-1^, respectively. Brillouin zone sampling utilized a 3×3×1 grid of K-points. Grimme’s DFT-D3 methodology was employed to account for the dispersion interactions.^5^

The adsorption energies (*E*_ads_) are calculated according to the equation:

*E*_ads_ = *E*_*Na_ - *E*_Na_ - *E*_Sub_

where *E*_Na_ and *E*_*Na_ indicate the energies before and after the adsorption of Na on the substrate, respectively. *E*_sub_ is the energy of clean surface.


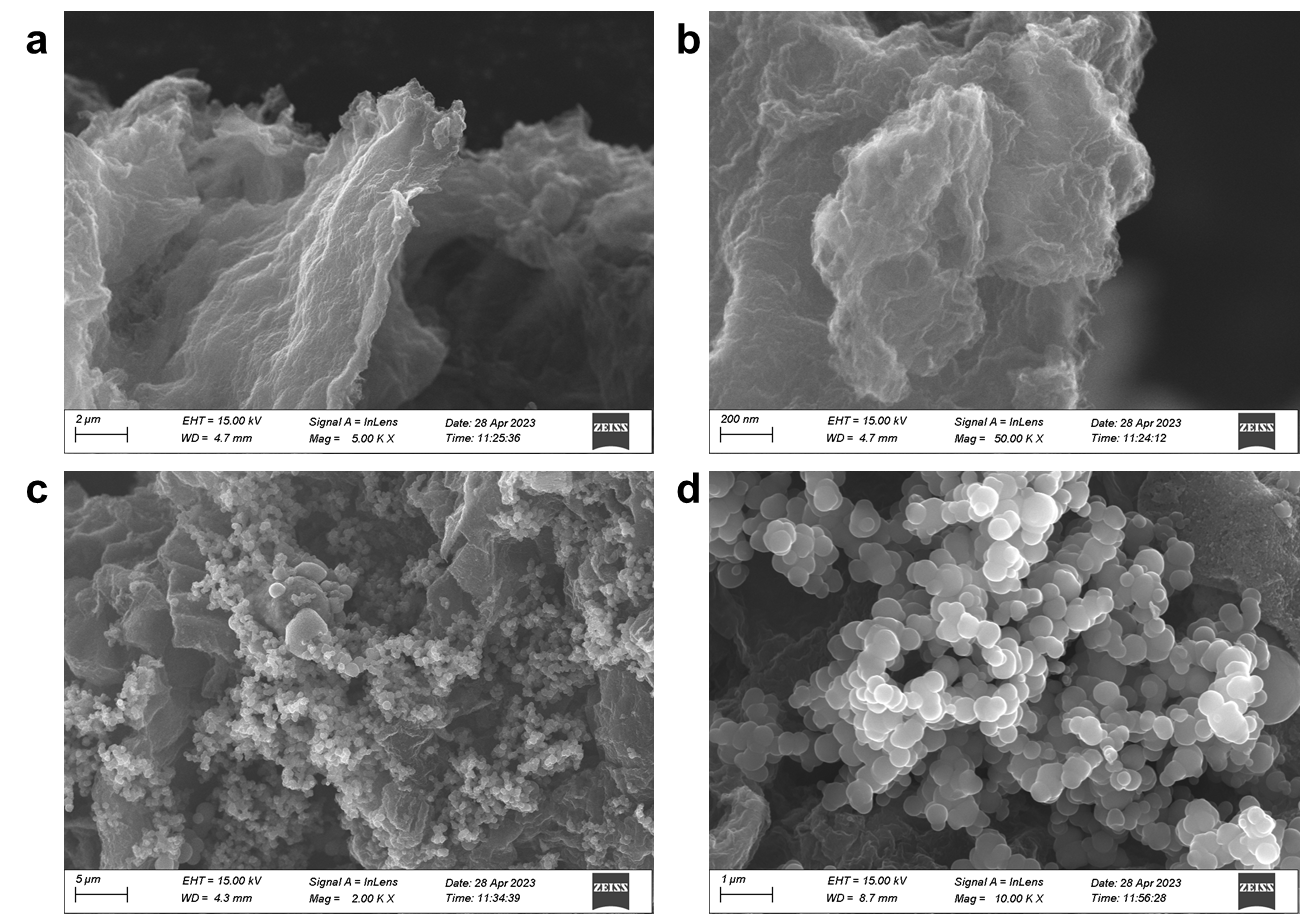


**Figure S1.** Morphology of rGO nanosheets (a-b) and Cu_3_SbS_3_@rGO composite (c-d).


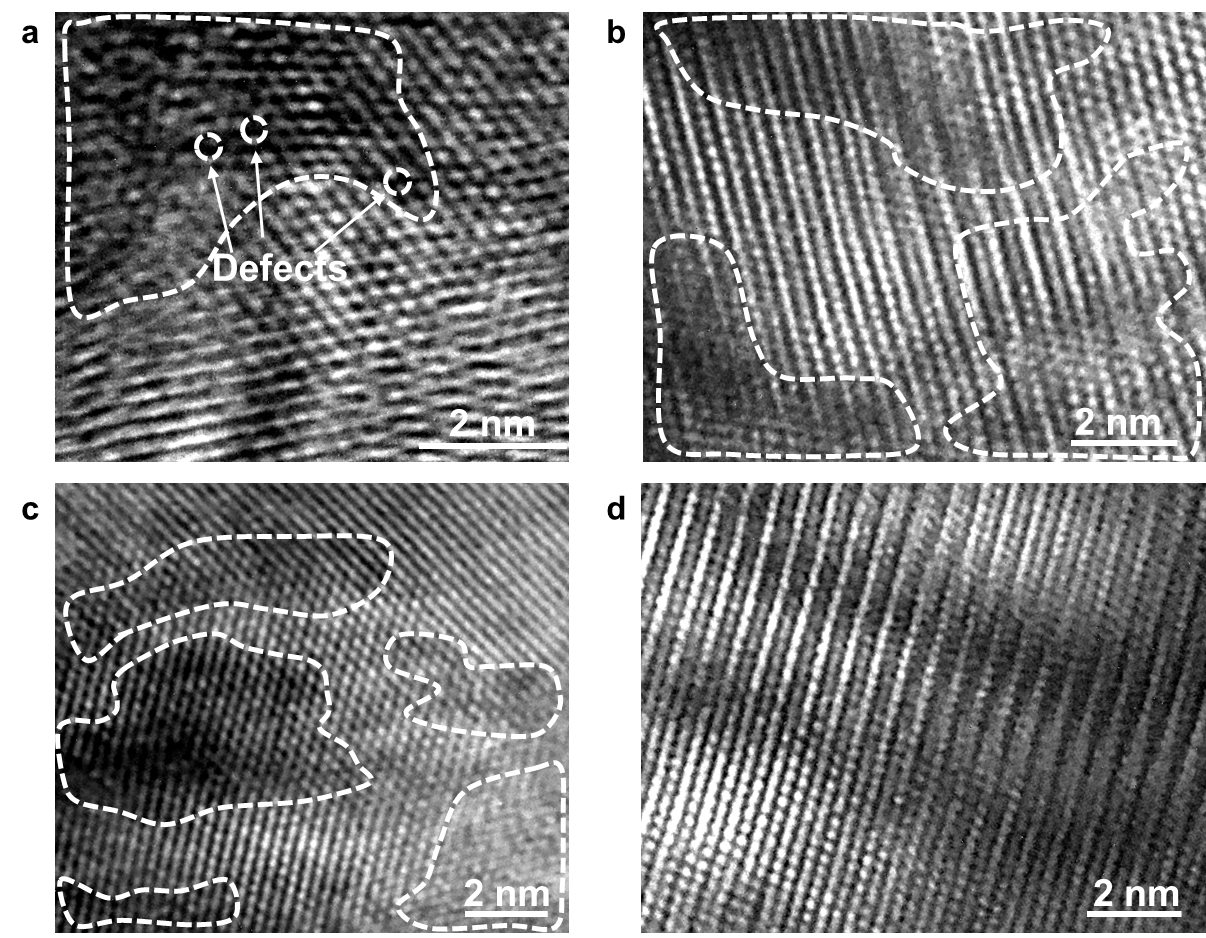


**Figure S2.** TEM images of the (444) planes for (a-c) Se-Cu_3_SbS_3_@rGO and (d) Cu_3_SbS_3_@rGO.


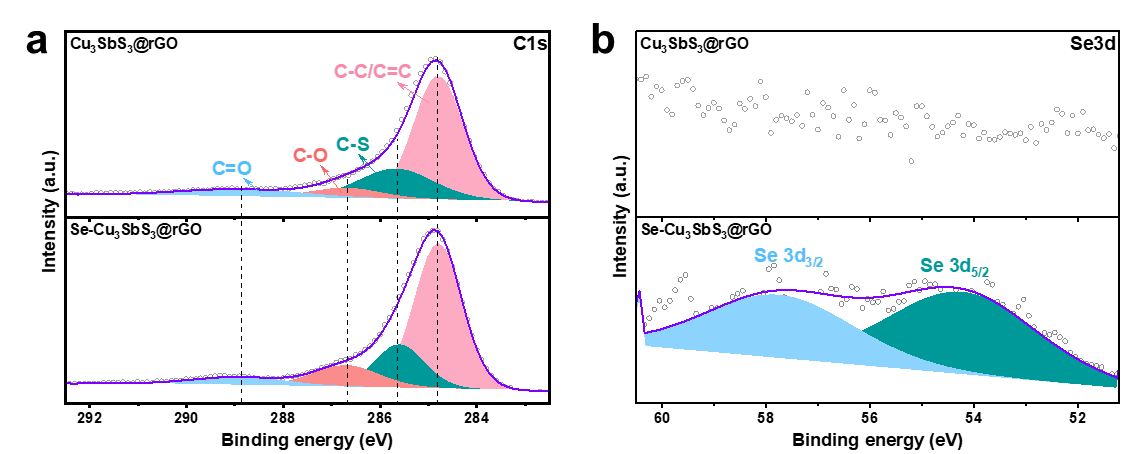


**Figure S3**. High-solution XPS spectra of the (a) C 1s and (b) Se 3d for Se-Cu_3_SbS_3_@rGO, and Cu_3_SbS_3_@rGO.


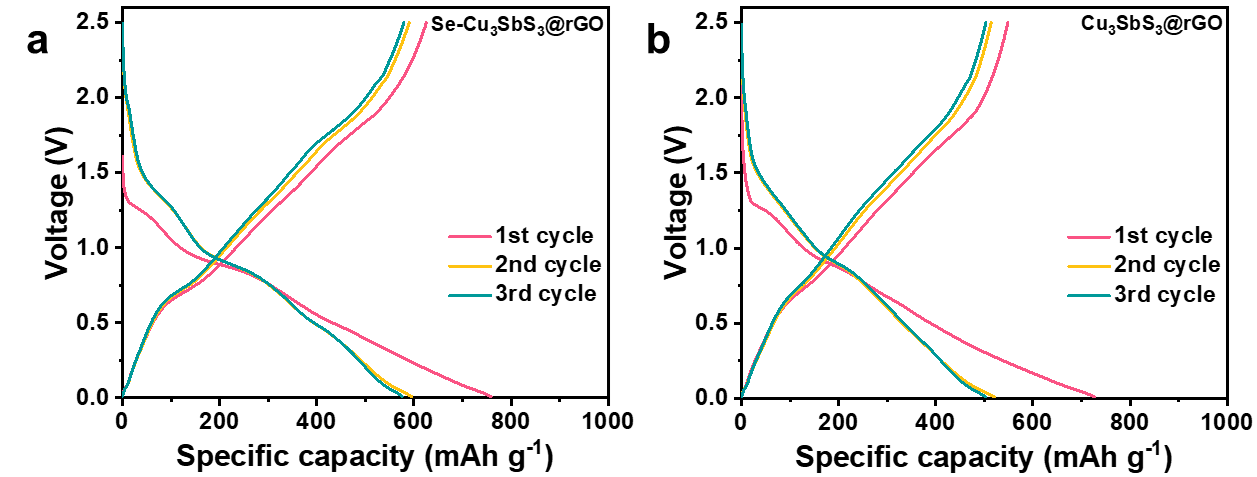


**Figure S4**. The first three charge-discharge profiles of (a) Se-Cu_3_SbS_3_@rGO and (b) Cu_3_SbS_3_@rGO.


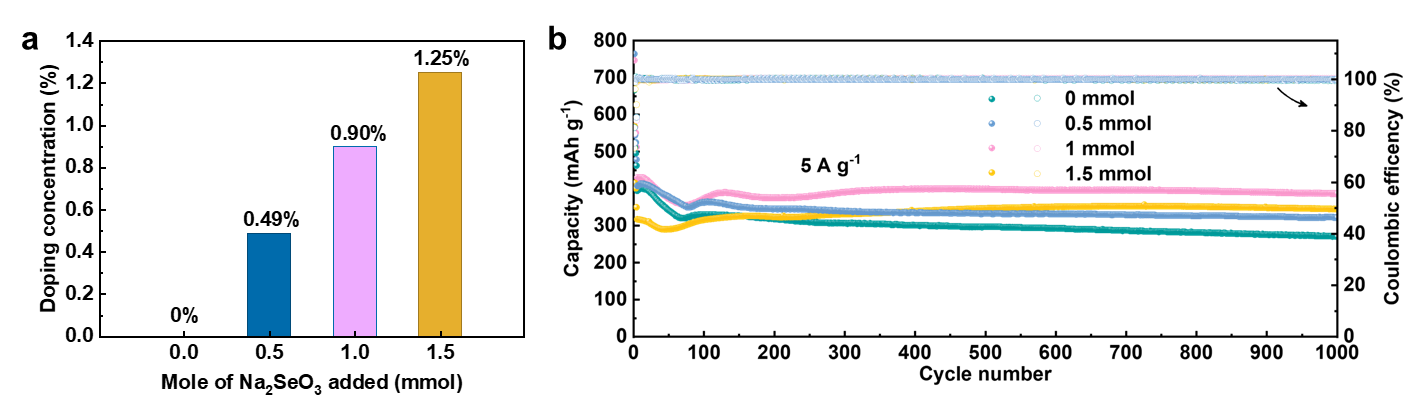


**Figure S5.** (a) ICP-OES analysis for the Se concentration in the Se-Cu_3_SbS_3_@rGO products synthesized with different Na_2_SeO_3_ amounts; (b) Cycling performance of Se-Cu_3_SbS_3_@rGO with different doping levels prepared by adding 0, 0.5, 1.0, and 1.5 mmol Na_2_SeO_3_ at 5 A g^-1^.

**Figure S6.** The charge-discharge curves of Cu_3_SbS_3_@rGO at different current densities**.**


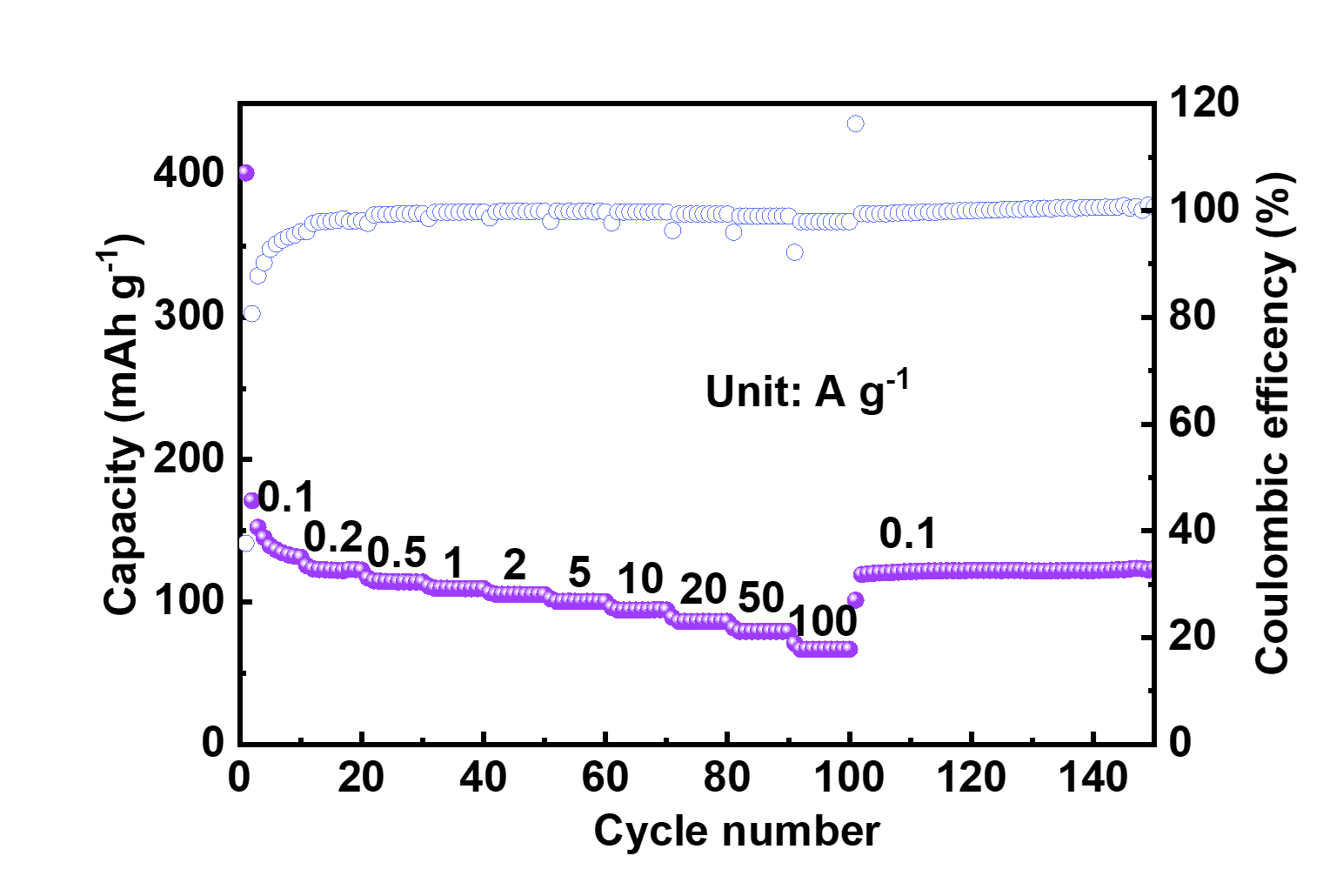


**Figure S7.** Rate performance of pure rGO.


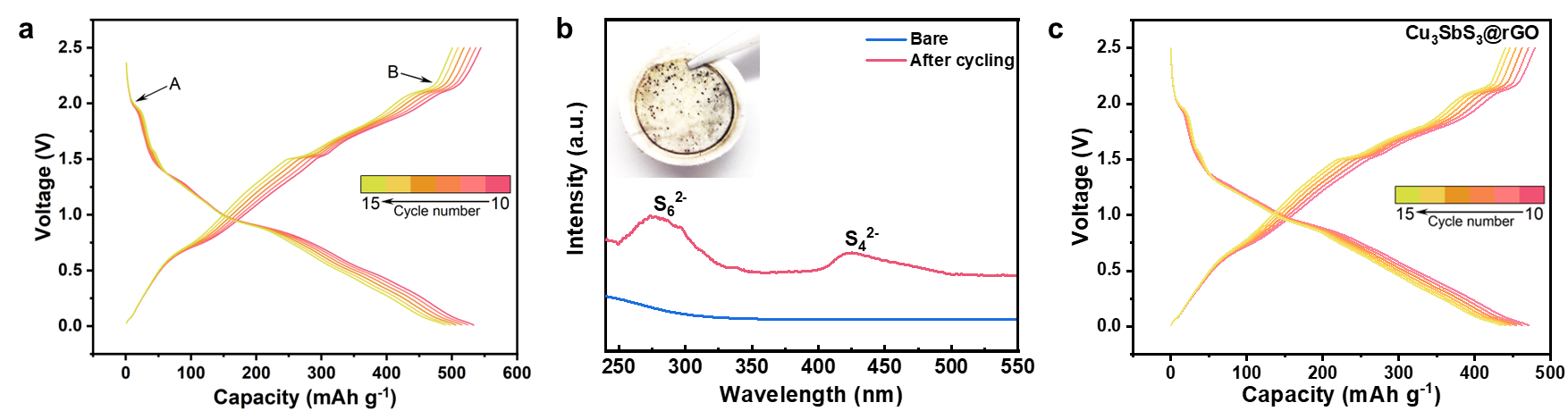


**Figure S8**. (a) The discharge-charge profiles of the 10th to 15th cycles of Se-Cu_3_SbS_3_@rGO anode. (b) UV-Vis tests for the separator after 30 cycles and bare separator; Insert in (b) is a photograph of the separator after 30 cycles. (c) The discharge-charge profiles of the 10th to 15th cycles of Cu_3_SbS_3_@rGO anode.


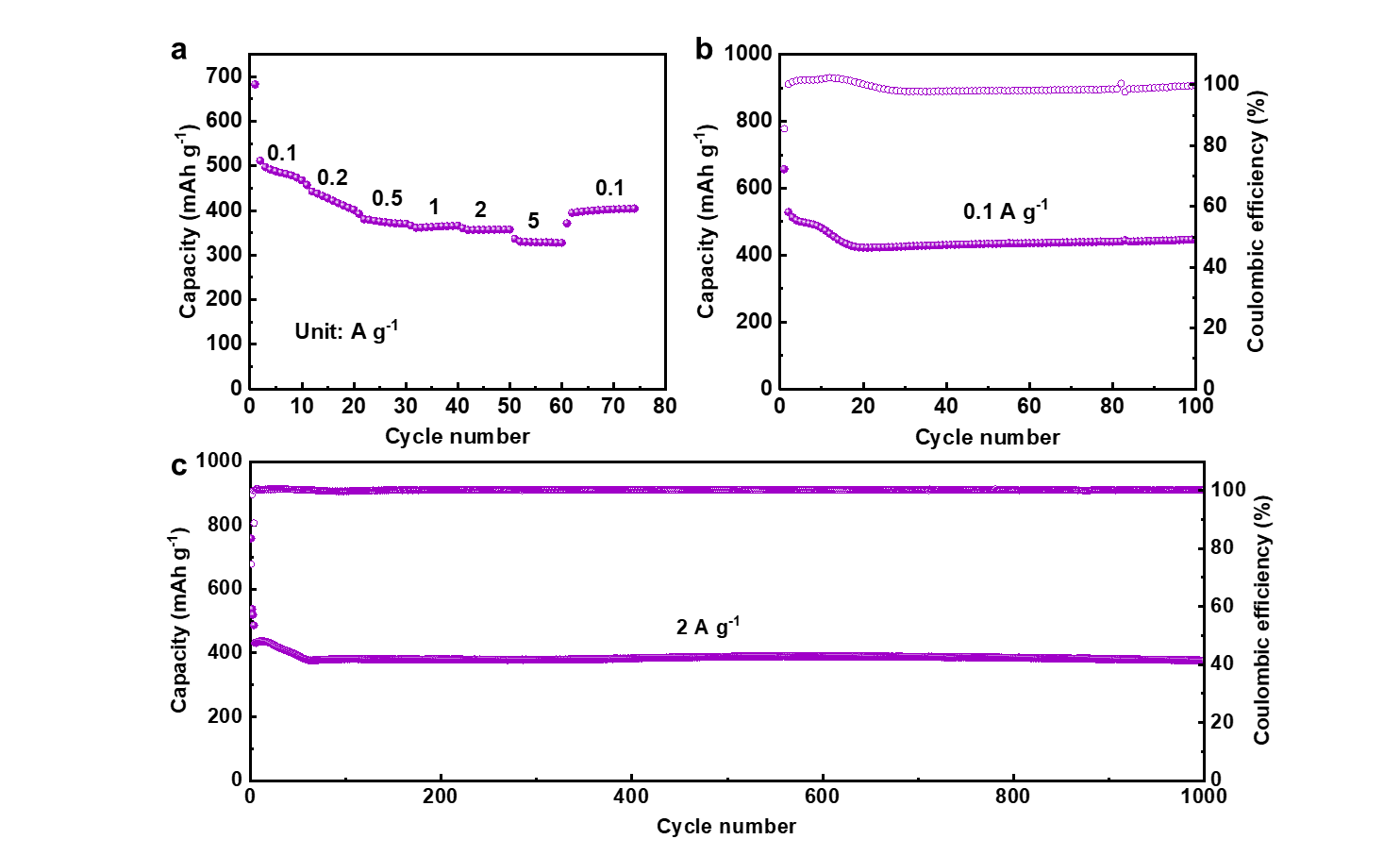


**Figure S9**. (a) Rate performance of Se-Cu_3_SbS_3_@rGO with a mass loading of 6 mg cm^-2^. Cycling performance of Se-Cu_3_SbS_3_@rGO with a mass loading of 6 mg cm^-2^ at (b) 0.1 A g^-1^ and (c) 2 A g^-1^.


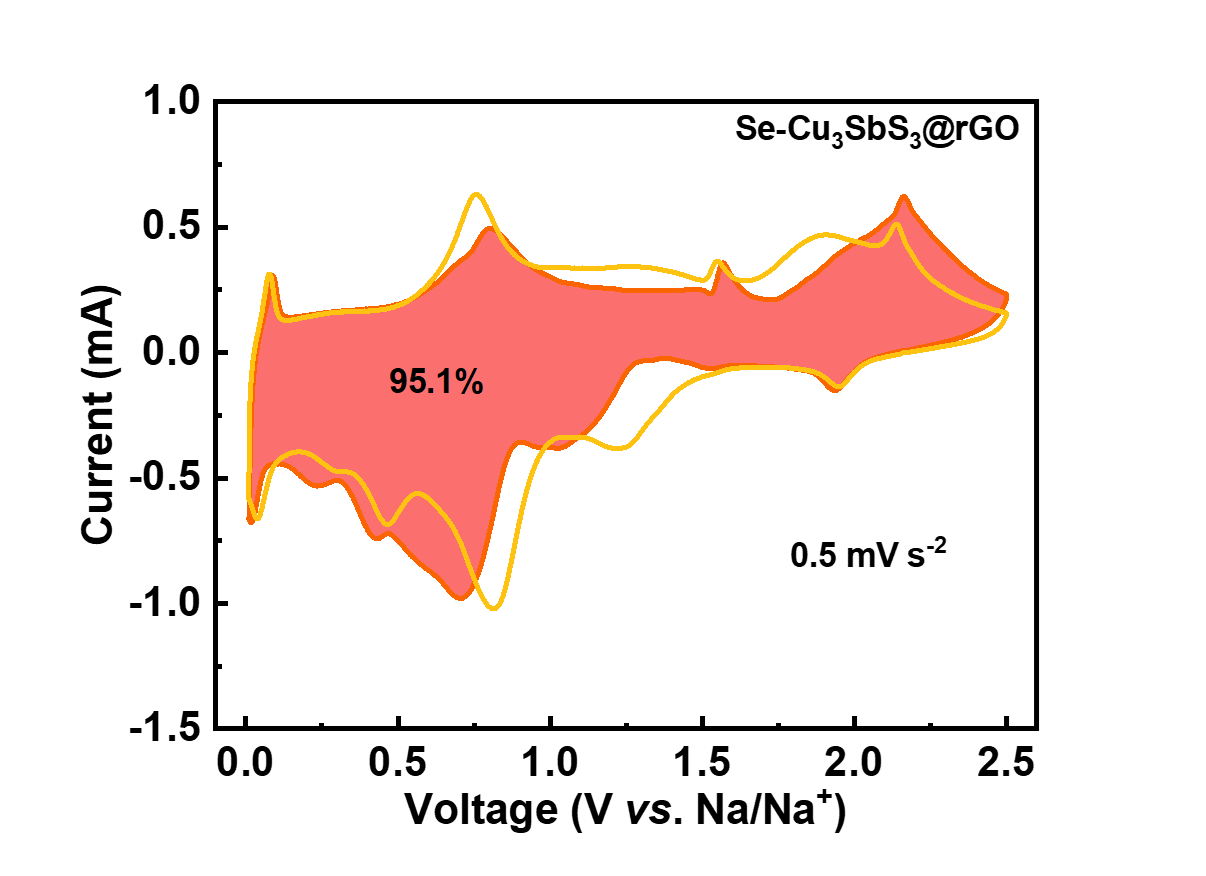


**Figure S10.** The detailed capacitive contribution of Se-Cu_3_SbS_3_@rGO electrode at 0.5 mV s^-1^.


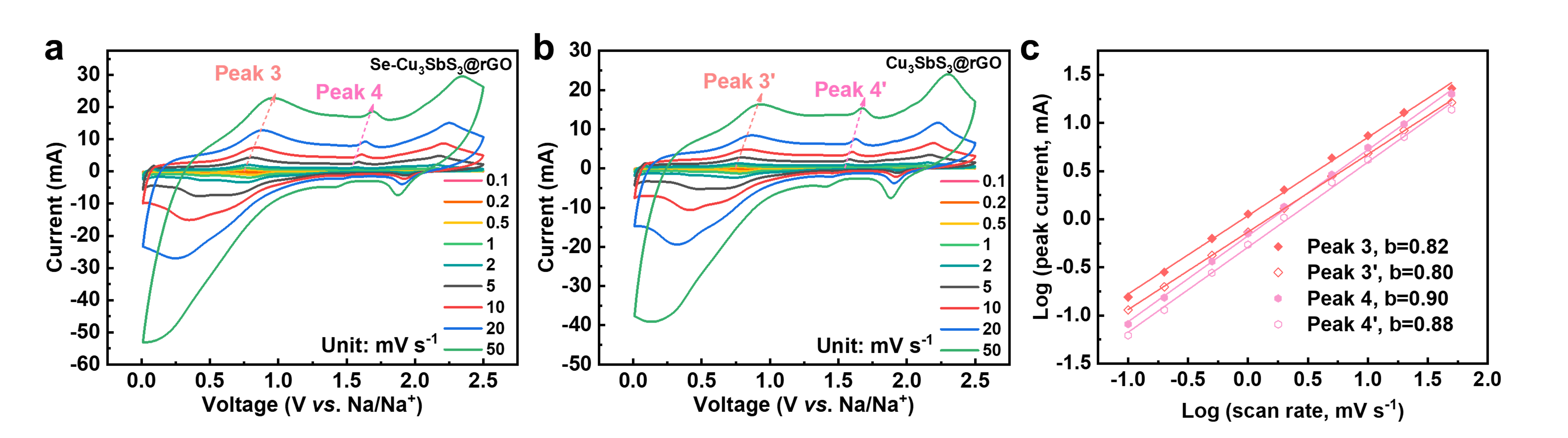


**Figure S11.** CV curves at different scan rates of (a) Se-Cu_3_SbS_3_@rGO and (b) Cu_3_SbS_3_@rGO. (c) Fitted b values of the marked peaks in (a) and (b).


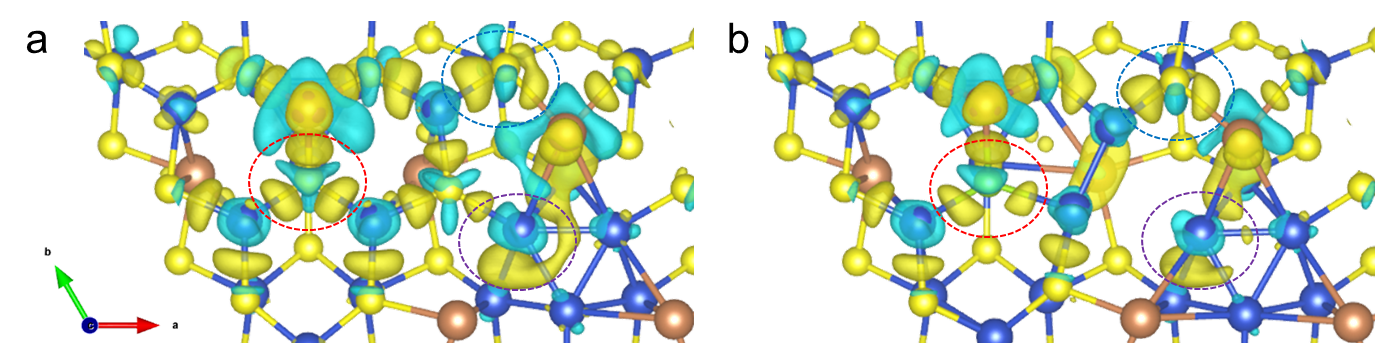


**Figure S12.** Charge density differences of (a) Cu_3_SbS_3_ and (b) Se-Cu_3_SbS_3_.

**Figure S13.** In-situ XRD curves of Se-Cu_3_SbS_3_@rGO anode during charge-dicharge process**.**


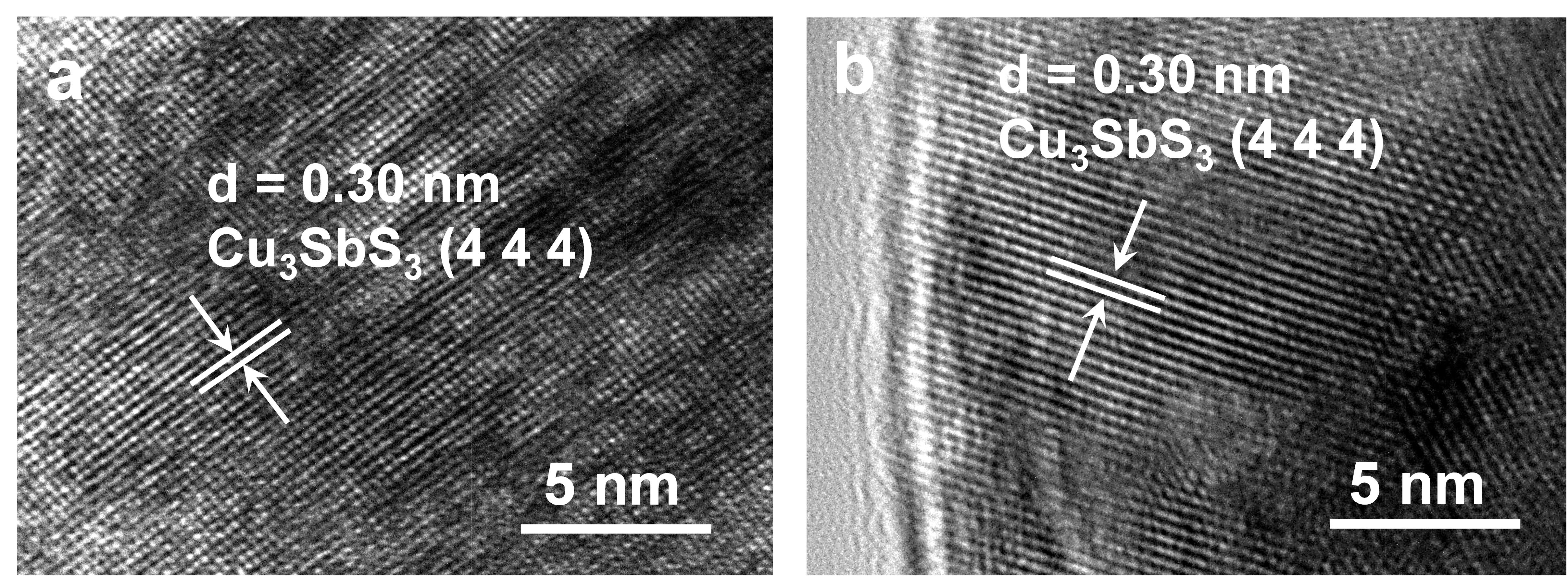


**Figure S14.** TEM image of Se-Cu_3_SbS_3_@rGO after (a) 50 cycles and (b) 1000 cycles.


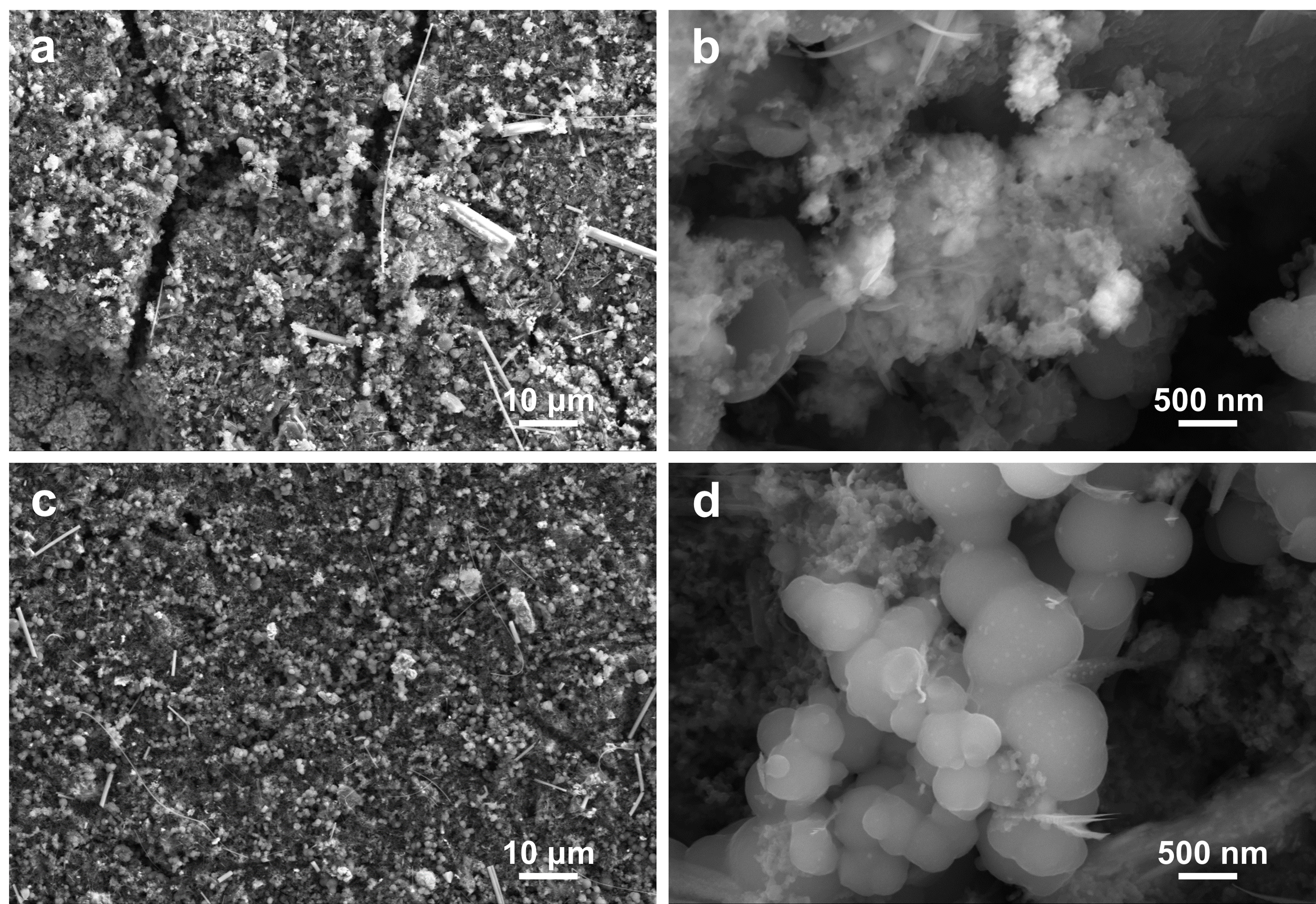


**Figure S15.** SEM images of (a, b) Cu_3_SbS_3_@rGO and (c, d) Se-Cu_3_SbS_3_@rGO electrodes after 1000 cycles at 5 A g^-1^.


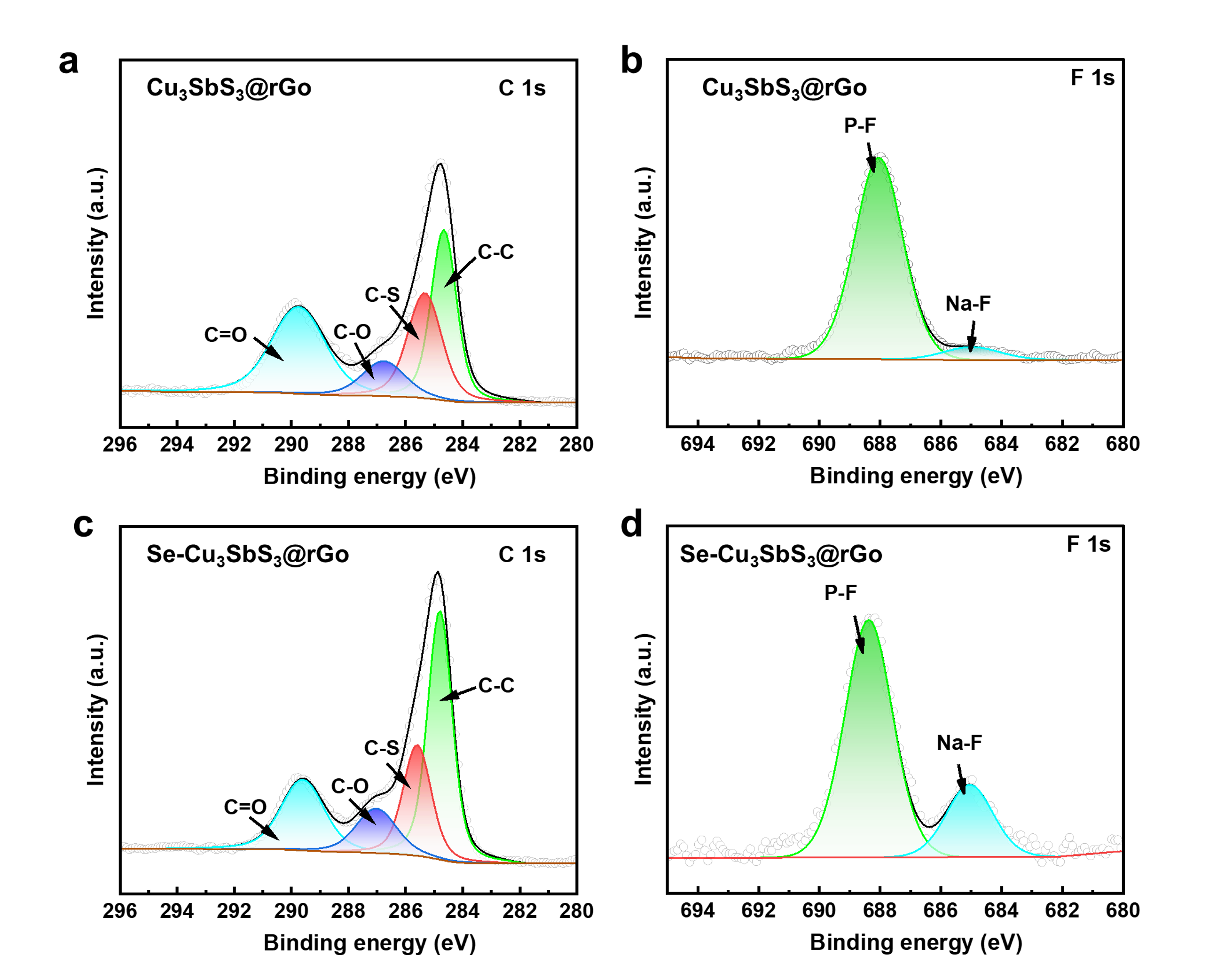


**Figure S16.** XPS analyses of Cu_3_SbS_3_@rGO (a, b) and Se-Cu_3_SbS_3_@rGO (c, d) electrodes after 1000 cycles.

**
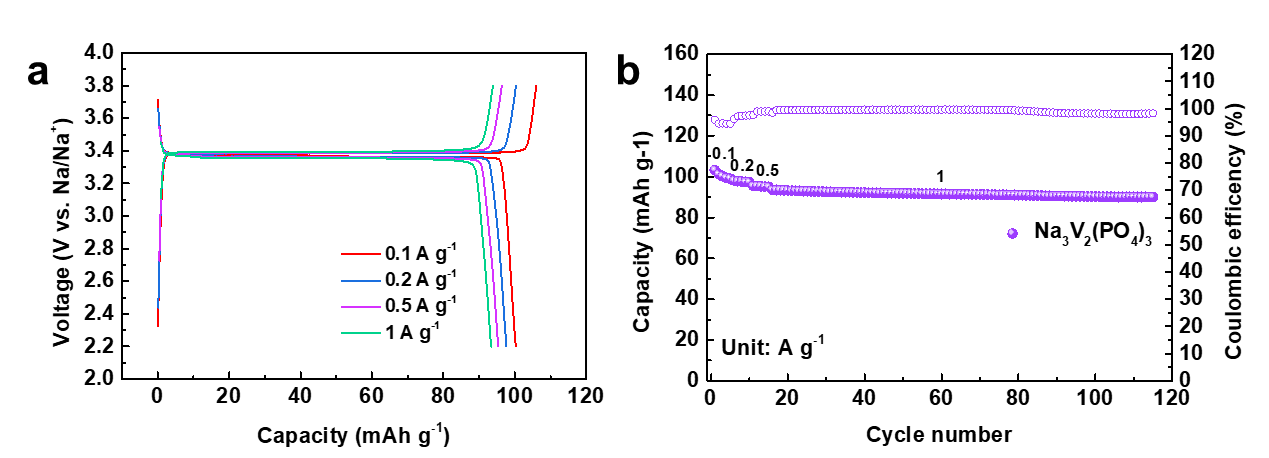
**

**Figure S17.** Charge-dischagre curves (a) and cycling performance (b) of Na_3_V_2_(PO_4_)_3_@C cathodes.


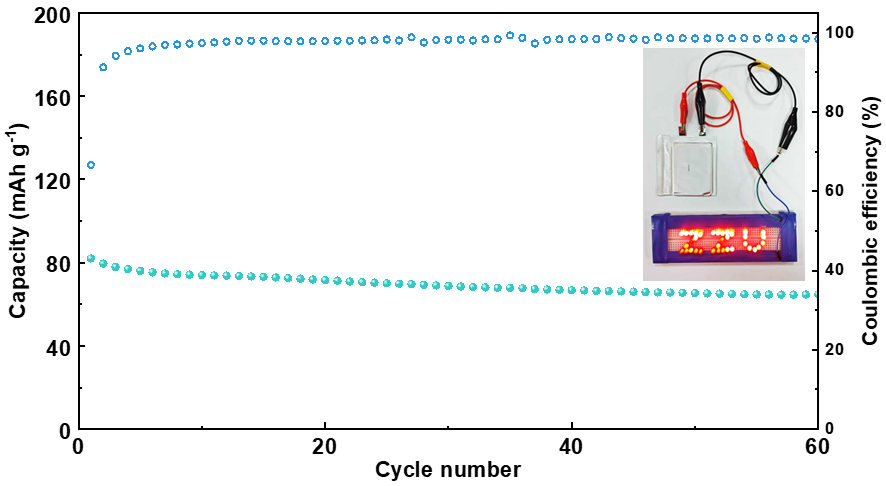


**Figure S18**. Cycling performance of the NVP@C||Se-Cu_3_SbS_3_@rGO pouch cell at 0.5 A g^-1^.

**Table S1.** Electrochemical performance for Se-Cu_3_SbS_3_@rGO compared to other reported advanced sulfide anodes in SIBs.

| **Sample** | **Rate performance** | **Cycling performance** | **Ref.** |
| --- | --- | --- | --- |
| **Se-Cu_3_SbS_3_@rGO** | 569.1 mAh g^-1^ at 0.1 A g^-1^  328.4 mAh g^-1^ at 20 A g^-1^ | 2 A g^-1^, 1000 cycles (~90.2%)  5 A g^-1^, 1000 cycles (~90.0%) | This work |
| **Cu_2_S@C nanoboxes** | 200 mAh g^-1^ at 0.1 A g^-1^  75 mAh g^-1^ at 2.5 A g^-1^ | 0.05 A g^-1^, 200 cycles (~39.8%) | [11] |
| **Cu_2_S@Carbon@MoS_2_** | 410 mAh g^-1^ at 0.1 A g^-1^  316 mAh g^-1^ at 2 A g^-1^ | 0.3 A g^-1^, 200 cycles (~80.4%) | [12] |
| **Cu_3_SnS_4_@CNT** | 409.7 mAh g^-1^ at 1 A g^-1^ 161.6 mAh g^-1^ at 20 A g^-1^ | 1 A g^-1^, 1000 cycles (84.5%) | [13] |
| **Sb_2_S_3_@NCR** | 592.5 mAh g^-1^ at 0.1 A g^-1^  148 mAh g^-1^ at 10 A g^-1^ | 1 A g^-1^, 1000 cycles (77%) | [14] |
| **Ti_3_C_2_-Sb_2_S_3_** | 470 mAh g^-1^ at 0.2 A g^-1^  370 mAh g^-1^ at 2 A g^-1^ | 1 A g^-1^, 500 cycles (70.7%) | [15] |
| **(Bi-Sb)_2_S_3_@N-C** | 688 mAh g^-1^ at 0.1 A g^-1^  251 mAh g^-1^ at 2 A g^-1^ | 2 A g^-1^, 1600 cycles (85%) | [16] |
| **M-Sb_2_S_3_@DC** | 702 mAh g^-1^ at 0.2 A g^-1^  366 mAh g^-1^ at 3 A g^-1^ | 0.5 A g^-1^, 100 cycles (65%) | [17] |

**Equation S1**:

The following Equations were used to calculate b values from the CV data: ^[6]^

$$i=av^{b}$$

$$\log\left( i \right)=blog\left( v \right)+log(a)$$

where, i is the peak current, ν is the scan rate. A b value of 0.5 indicates a diffusion-controlled process, while a b value of 1.0 represents the capacitive-dominated process.

**Equation S2**:

The following Equations were used to calculate capacitive contributions from the CV data: ^[7]^

$$i=k_{1}v+k_{2}v^{1/2}$$

$$i/v^{1/2}=k_{1}v^{1/2}+k_{2}$$

Where, *k*_1_*v* represents the capacitive-dominant process, *k*_1_*v*^1/2^ represents the diffusion-dominant reactions.

**Equation S3**:

The following Equation was used to calculate the Na^+^ diffusion coefficients from the GITT data:^[8]^

$$D_{{Na}^{+}}=\frac{4}{\pi\tau}({\frac{m_{B}V_{m}}{M_{B}S})}^{2}{\cdot\left( \frac{\Delta E_{s}}{\Delta E_{t}} \right)}^{2}=\frac{4L^{2}}{\pi\tau}{\cdot\left( \frac{\Delta E_{s}}{\Delta E_{t}} \right)}^{2}$$

where m_B_ is the mass of the active materials, 𝑉_M_ is the molar volume of the active materials, M_B_ is the molar mass of the active materials, S is the contact area between the active materials and electrolyte, τ is the relaxation time, t is the time of duration current pulse, and ΔE_s_ is the potential change of steady state caused by current pulse, ΔE_t_ is the potential change during the constant current pulse after eliminating the iR drop. L is the diffusion length of sodium ions. For a compact electrode, L equals to the thickness of the electrode.

**Equation S4**:

The following Equations were used to calculate the Warburg factors from the EIS data: ^[9]^

$$\omega=2\pi f$$

$$Z'=R+\sigma\omega^{-1/2}$$

in which f is the frequency.

**Equation S5**:

The following Equation was used to calculate the Na^+^ diffusion coefficient from the EIS data:^[10]^

$$D_{{Na}^{+}}=\frac{R^{2}T^{2}}{2A^{2}n^{2}F^{4}C^{2}\sigma^{2}}$$

in which A is the electrode area, n is the number of electronic transfers per molecule, C is the molar concentration of Na^+^, R is the gas constant (8.314 J mol^-l^ K^-1^), T is the absolute temperature (298 K), F is the Faraday constant (96500 C mol^-1^), σ is the Warburg factor obtained from the line slope of Z′ versus ω^-1/2^ (Equation S4).

**References**

[1] G. Kresse, J. Hafner, Phys Rev B 1993, 47, 558.

[2] G. Kresse, J. Hafner, Phys Rev B 1994, 49, 14251.

[3] J. P. Perdew, K. Burke, M. Ernzerhof, Phys Rev Lett 1996, 77, 3865.

[4] G. Kresse, D. Joubert, Phys Rev B 1999, 59, 1758.

[5] P. E. Blöchl, Phys Rev B 1994, 50, 17953.

[6] Y. Liang, Z. Wang, Z. Xu, S. Li, H. Luo, C. Xu, X. Cui, Appl Surf Sci 2024, 651, 159234.

[7] Y. Xu, X. Liu, H. Su, S. Jiang, J. Zhang, D. Li, Energy & Environmental Materials 2022, 5, 627–636.

[8] H. He, H. Zhang, D. Huang, W. Kuang, X. Li, J. Hao, Z. Guo, C. Zhang, Adv. Mater. 2022, 34, 2200397.

[9] D. Wang, Y. Chao, K. Guo, Z. Wang, M. Yang, J. Zhu, X. Cui, Q. Xu, Adv. Funct. Mater. 2024, 2405642.

[10] K. Qian, L. Li, D. Yang, B. Wang, H. Wang, G. Yuan, J. Bai, S. Ma, and G. Wang, Adv. Funct. Mater. 2023, 33, 2213009.

[11] D Zhao, B Du, T Li, C Xu, Y Li, Q Yin, F Wei, J Qi, Y Sui, Appl. Surf. Sci. 2024, 674, 160910.

[12] Y Fang, D Luan, Y Chen, S Gao, X W Lou, Angew. Chem. Int. Ed. 2020, 59, 7178 –7183.

[13] Y Jin, H Yoo, H Seong, J H Moon, G Kim, T Jung, Y Myung, W Lee, S Kim, J Choi, Chem. Eng. J. 2025, 503, 158461.

[14] D Li, J Li, H Liu, G Li, G Milinevsky, L Wang, W Han, Chem. Eng. J. 2023, 477, 147045.

[15] H Wang, X Song, M Lv, S Jin, JXu, X Kong, X Li, Z Liu, X Chang, W Sun, J Zheng, X Li, Small 2022, 18, 2104293.

[16] L Yang, L Guo, D Yan, Y Wang, T Shen, D Li, M E Pam, Y Shi, H Y Yang, ACS Nano 2023, 17, 6754−6769.

[17] P Ge, L Zhang, W Zhao, Y Yang, W Sun, X Ji, Adv. Funct. Mater. 2020, 30, 1910599.
